# Supplementary material for: Interactome Analysis and Docking Sites of MutS Homologs Reveal New Physiological Roles in Arabidopsis thaliana
Source: Molecules. 2019 Jul 8;24(13):2493. doi: 10.3390/molecules24132493 (PMC6651420; doi:10.3390/molecules24132493)
Supplement: Supplementary file 1 [file molecules-24-02493-s001.pdf]

SUPPLEMENTARY MATERIAL

Interactome Analysis and Docking Sites of MutS Homologs Reveal new Physiological Roles in *Arabidopsis Thaliana*

Mohamed Ragab AbdelGawwad [1,*], Aida Marić [2], Abdullah Ahmed Al-Ghamdi [3] and Ashraf A. Hatamleh [3]

1. Genetics and Bioengineering, Faculty of Engineering and Natural Sciences, International University of Sarajevo, Sarajevo 71210, Bosnia and Herzegovina
2. Centre for Research in Agricultural Genomics, UAB-Edifici CRAG, Cerdanyola, Barcelona 08193, Spain;
3. Department of Botany and Microbiology, College of Sciences, King Saud University, Riyadh 11451, Saudi Arabia

* Correspondence: mragab@ius.edu.ba; Tel.: +3873-3957-203

```
MSH1_ARATH      -----------------------------------------------------MHW----
MSH4_ARATH      ------------------------------------------------------------
MSH5_ARATH      ------------------------------------------------------------
MSH6_ARATH      EKTEWVVGEKSGDRFNRLKRGASALRKVVTDSDDDVEMGNVEEDKSDGDDSSDEDWGKNV
MSH7_ARATH      ----------------------------VK----------VDDRDCSGERS-REDVVPLN
MSH2_ARATH      ------------------------------------------------------------
MSH3_ARATH      ------------------------------------------------------------

MSH1_ARATH      -------------------------IATRNAVVSFPKWRFFFRSSYR-------TYSSLK
MSH4_ARATH      ------------------------------------------------------------
MSH5_ARATH      ------------------------------------------------------------
MSH6_ARATH      GKEVCESEEDDVELVDENEMDEEELVEEKDEETSKVNRVSKTDSRKRKTSEVTKSGG---
MSH7_ARATH      DSSLCMKANDVIPQFRSNNGKT----QERNHAFSFS---GRAE--LRSVEDIGVDGDVPG
MSH2_ARATH      ------------------------------------------------------------
MSH3_ARATH      --------------------------------------MGKQK-----QQTIS-RFFAPK

MSH1_ARATH      PSSPILLNRRYSEG---ISCLRDGKSLKRI----TTASKKVKTSSDVL------------
MSH4_ARATH      ------------------------------------------------------------
MSH5_ARATH      ------------------------------------------------------------
MSH6_ARATH      -EK----------K----SKTDTGTILKGFKASVVEPAKKIGQADRVVKG----LEDNVL
MSH7_ARATH      PETPGMRPRASRLK----RVLEDEMTFKEDKVPVLDSNKRLKMLQDPVCG----EKK---
MSH2_ARATH      ------------------------------------------------------MEGNFE
MSH3_ARATH      PKSPTHEPNPVAESSTPPPKISATVSF--------SPSKRKLLSDHLAAASPKKPKLSPH

MSH1_ARATH      ------------------------------------------------------------
MSH4_ARATH      ------------------------------------------------------------
MSH5_ARATH      ------------------------------------------------------------
MSH6_ARATH      MAPSRRQISGRSPLVNQQRQITSFFGKSASSSSSPSPS----------PSPSLSNKKTPK
MSH7_ARATH      --------------MQRQRSILSFFQKPTAATTKGLVSGDAASGGGGSGGPRFNVKEGDA
MSH2_ARATH      ------------------------------------------------------------
MSH3_ARATH      ------------------------------------------------------------

MSH1_ARATH      ------------------------------------------------------------
MSH4_ARATH      ------------------------------------------------------------
MSH5_ARATH      ------------------------------------------------------------
MSH6_ARATH      SNNPNPKSPSPSPSPPKKTPKLNPNPSSNLPARSPSPGPDTPSPVQSKFKKPLLVIGQTP
MSH7_ARATH      KGDAS--------------VRFAVSKS-----VDEVRGTDTPPE---KVPRRVLPSGFKP
MSH2_ARATH      ------------------------------------------------------------
MSH3_ARATH      ------------------------------------------------------------

MSH1_ARATH      ------------------------------------------------------------
MSH4_ARATH      ------------------------------------------------------------
MSH5_ARATH      ------------------------------------------------------------
MSH6_ARATH      SPPQSVVITYGDEVVGKQVRVYWPLDKKWYDGSVTFYDKGEGKHVVEYEDGEEESLDLGK
MSH7_ARATH      AE-----------SAGDASSLFSNIMHKF-------------------------------
MSH2_ARATH      ------------------------------------------------------------
MSH3_ARATH      ------------------------------------------------------------
```

```
MSH1_ARATH      TDKDL-------SHLVWWKE----RLQTCKKPSTLQLIERLMYTNLLGLDPSLRNGSLKD
MSH4_ARATH      ------------------------------------------------------------
MSH5_ARATH      ------------------------------------------------------------
MSH6_ARATH      DGDALARFGARDSEKFRFLGV-DRRDAKRRRPTDENYDPRT-----LYLPPDFV---KKL
MSH7_ARATH      --------EVNEGTKFEWLESSRIRDANRRRPDDPLYDRKT-----LHIPPDVF---KKM
MSH2_ARATH      EQNKLPELKLDAKQAQGFLS----------------------------------------
MSH3_ARATH      TQNPVPDPNLHQRFLQRFLE-----------PSPEEYVPET----------SSS---RKY

MSH1_ARATH      GNLNWEMLQFKSRFPREVLLCRVGEFYEAIGIDACILVEYAGLNPFGGLRSDSIPKAGCP
MSH4_ARATH      ------------------------------------------------------------
MSH5_ARATH      ------------------------------------------------------------
MSH6_ARATH      TGGQRQWWEFKAKHMDKVVFFKMGKFYELFEMDAHVGAKELDIQYMKG-EQ---PHCGFP
MSH7_ARATH      SASQKQYWSVKSEYMDIVLFFKVGKFYELYELDAELGHKELDWKMTMS-GVGKCRQVGIS
MSH2_ARATH      ---------FYKTLPNDTRAVRFFDRKDYYTAHGENSVFIAKTYYHTT-T----------
MSH3_ARATH      TPLEQQVVELKSKYPDVVLMVEVGYRYRFFGEDAEIAARVLGIYAHMD-HN--FMTASVP

MSH1_ARATH      IMNLRQTLDDLTRNGYSVCIVEEVQG------PTPARSRKGRFIS---------------
MSH4_ARATH      ------------------------------------------------------------
MSH5_ARATH      ------------------------------------------------------------
MSH6_ARATH      EKNFSVNIEKLVRKGYRVLVVEQTET------PDQLEQRRKETGSKDKVVKREVCAVVTK
MSH7_ARATH      ESGIDEAVQKLLARGYKVGRIEQLET------SDQAKARGANT-----IIPRKLVQVLTP
MSH2_ARATH      ------ALRQLGSGSNALSSVSISRNMFETIARDLLLERNDHTVE----LYEGSGSNWRL
MSH3_ARATH      TFRLNFHVRRLVNAGYKIGVVKQT------ETAAIKSHGANRTGP----FFRGLSALYTK

MSH1_ARATH      -----------GHA--------HPGSPYVYGLVGVD---------HDLDFPDPMPVVGIS
MSH4_ARATH      ---------ME---DDG-----GERSSFVAG-----------------LIENRAKEVGMA
MSH5_ARATH      ---------------------------------MEEMEDTETEPQVYMACIQHGRRVGVS
MSH6_ARATH      GTL-----------TDGEMLLTNPDASYLMALTEGGES---------LTNPTAEHNFGVC
MSH7_ARATH      STA-----------SEGNI---GPDAVHLLAIKEIKM-----------ELQKCSTVYGFA
MSH2_ARATH      --------VKTGS--PGNI---GSFEDVLFA--NNEMQDTPVVVSIFPSFHDGRCVIGMA
MSH3_ARATH      ATLEAAEDISGGCGGEEGF---GSQSNFLVCVVDERVKSETLGCGIEMSFDVRVGVVG--
                                                                         *

MSH1_ARATH      R---SARGYCMISIFETMKAYS-----------------------------------LDD
MSH4_ARATH      AFDLRSASLHLSQYIETSSSYQNT--KTLLRFYDPSVIIVPPNKLAA------------D
MSH5_ARATH      YYDCSVRQLHVLEFWEED-CSDFTLINMVKYQAKP-SIIYASTKS---EESFVAALQQND
MSH6_ARATH      LVDVATQKIILGQFKDDQDCSALSC---LLSEMRPVEIIKPAKVLSYATERTIVRQTR-N
MSH7_ARATH      FVDCAALRFWVGSISDDASCAALGA---LLMQVSPKEVLYDSKGLSREAQKALRKYTL-T
MSH2_ARATH      YVDLTRRVLGLAEFLDDSRFTNLES---SLIALGAKECIFP-------------AE---S
MSH3_ARATH      -VEISTGEVVYEEFNDNFMRSGLEA---VILSLSPAELLLGQPLS-QQTEKFLVAH---A
                            .  :

MSH1_ARATH      GLTEEALVTKLRTRRCHHLFLHASLRHNASGTCRWGEFGEGGLLWG--ECSS----RNFE
MSH4_ARATH      GM---VGVSELV-DRCY------------STVRKV--------VFA------------RG
MSH5_ARATH      GTDETTMVKLVK-SSTF------------SYEQAWHRL-----VYLRVTGMD------DG
MSH6_ARATH      -PLVNNLVP--L-SEFW------------DSEKTIYEVG---IIYKRINCQPSSAYSSEG
MSH7_ARATH      GSTAVQLAP--V-PQVM------------GDTDAAGV---------------RNIIESNG
MSH2_ARATH      GKS----------NECK------------SLYDSLERCA---VMIT---------ERKKH
MSH3_ARATH      GPTSNVRVERAS-LDCF------------SNGNAVDEVI---------------------

MSH1_ARATH      WFEGDTLSELLSRVKDV--------------YGLD-------------------------
MSH4_ARATH      CFDDTKGAVLIQN-----LAAEEP-----LALGLDTYYKQHYLSLAAAAA-----TIKWI
MSH5_ARATH      LNIKERICYLSSMMDVG-----------------------SEVQVRVSGG---LLAILES
MSH6_ARATH      KILGDGSSFLPKMLSEL-ATEDKNG----------------SLALSALGG-----AIYYL
MSH7_ARATH      YFKGSSESWNCA------VDGLNEC----------------DVALSALGE-----LINHL
MSH2_ARATH      EFKGRDLDSDLKRLVKGNIEPVRDLV-----SGFD-------LATPALG---ALLSFSEL
MSH3_ARATH      --------SLCEKISAGNLEDDKEMKLEAAEKGMSCLTVHTIMNMPHLTVQALALTFCHL

MSH1_ARATH      -----------------------------------DEVSFRNVNVPS-KNRPRPLHLGTA
MSH4_ARATH      EAEK--GVIVTN----H---SLTVTFNGSFDHMNIDATSVENLELIDPFHN---ALLGTS
MSH5_ARATH      E--RIVETLEQNESGSASIAIDSVMEVPLNKFLKLDAAAHEALQIFQTDKHPSHMGIGRA
MSH6_ARATH      RQAFLDESLLR-FAKFESLPYCDFSNVNEKQHMVLDAAALENLEIFENSRN--------G
MSH7_ARATH      SRLKLEDVLKH-G---DIFPYQVY-----RGCLRIDGQTMVNLEIFNNSCD--------G
MSH2_ARATH      L---SNEDNYG---------NFTIRRYDIGGFMRLDSAAMRALNVMESKTD--------A
MSH3_ARATH      KQFGFERILYQ---------GASFRSLSSNTEMTLSANTLQQLEVVKNNSD--------G
                                                   .  :   ::: .            .

MSH1_ARATH      TQIGALPTEGIPCLLKVLLPSTC-SGLPSLYVRDLLLNPPAYDIALKIQETCKLMSTVTC
MSH4_ARATH      NKKRSLFQ---------MFKTT-KTAGGTRLLRANL-LQPLKDIETINTR----LDCLDE
MSH5_ARATH      KEGFSVFG---------MMNKC-ATPMGRRLLRSWF-MRPILDLEVLDRR----LNAISF
MSH6_ARATH      GYSGTLYA---------QLNQC-ITASGKRLLKTWL-ARPLYNTELIKER----QDAVAI
MSH7_ARATH      GPSGTLYK---------YLDNC-VSPTGKRLLRNWI-CHPLKDVESINKR----LDVVEE
MSH2_ARATH      NKNFSLFG---------LMNRTCTAGMGKRLLHMWL-KQPLVDLNEIKTR----LDIVQC
MSH3_ARATH      SESGSLFH---------NMNHTL-TVYGSRLLRHWV-THPLCDRNLISAR----LDAVSE
                    ::            :     :      ::  .   *  :      .     . :
```

```
MSH1_ARATH      SIPEFTCVSSA----KLVKLLEQR----EANYIEFCRIKNVL------------------
MSH4_ARATH      LMSNEQLFFGLSQ--VLRKF-------PKETDRVLCHFCFKP---------------KKV
MSH5_ARATH      FISSVELMASLRET--LKS--------VKDISHLLKKFNSPTSLCTSNDWTAFLKSISAL
MSH6_ARATH      LRGENLPYSLEFR-KSLSR--------LPDMERLIARMFSSI-----------EAS-GRN
MSH7_ARATH      FTANSESMQ-ITG-QYLHK--------LPDLERLLGRIKSSV-----------RSS-ASV
MSH2_ARATH      FVEEAGLRQDLRQ--HLKR------ISD------VERLLRSL-----------ERRRGGL
MSH3_ARATH      ISACMGSHSSSQLSSELVEEGSERAIVSPEFYLVLSSVLTAM-----------SRSSDIQ
                                *                 .  .

MSH1_ARATH      -DDVLHMHRHAE--LVEILKLLMD------------PTWVATGLKID--FDTFVNECHWA
MSH4_ARATH      TEAVIGFENTRK--SQNMISS----------IILLKTALDALPILAK-----------VL
MSH5_ARATH      LHVNKIFEVGVSESLREHMR----RFNL--------DIIEKAGLCISTELD---------
MSH6_ARATH      GDKVVLYEDTAKKQVQEFISTLRGCETM---------------AEACSSLRA-I----LK
MSH7_ARATH      LPA-LLGKKVLKQRVKAFGQIVKGFRSG---------------I------DL-L----LA
MSH2_ARATH      QHIIKLYQSTIR--LP-FIKTAMQQYTGEFASLISERYLKKLEALS-------------D
MSH3_ARATH      RGITRIFHRTAK--ATEFIA-VME------AILLAGKQIQRLGIKQDSEMRS-MQSATVR
                       .

MSH1_ARATH      SDTIGEMISLDENESHQNVSKCDNVPN------EFFYD----MESSWRGRVKGIHIEEEI
MSH4_ARATH      KDAKCFLLANVYKS----VCENDRYASIRKKIGEVIDDDVLHARVPFVARTQQCFALKAG
MSH5_ARATH      -----YVYELVIGV---------------------IDVTRSKE----RGYQ---TLVKEG
MSH6_ARATH      HDTSRRLLHLLTPG----QS-LPNISSSIKYFKDAFDWVEAHN----SGRV----IPHEG
MSH7_ARATH      LQKESNMMSLLYKL----------------------------------------------
MSH2_ARATH      QDHLGKFIDLVECS----VD-LDQLE---------------------NGEY---MISSS-
MSH3_ARATH      STLLRKLISVISSP----VV-VDNAGKLL----SALNK-EAAV----RGDLLDILITSSD
      .

MSH1_ARATH      TQVE-KSAEALSLAVAEDFHPIISRIKATTASLGGPKGEIA--YAREHESVWFKGKRFTP
MSH4_ARATH      IDGFLDIARRTFCDTSEAIHNLASKYREEFN-LPNLKLP------------FNNRQG---
MSH5_ARATH      FCAELDELRQIYEELPEFLQEVSAMELEHFPHLHKEKLPPCI--------VYIQQIGYLM
MSH6_ARATH      ADEEYDCA----CKTVEEF--------ESSLKKHLKEQRKLLG---------DASINYVT
MSH7_ARATH      ------------C-----------------------KLPILVG---------KSG-----
MSH2_ARATH      YDTKLASLKD----QKELL--------EQQIHELHKKTAIELDLQVDKALKLDKAAQFGH
MSH3_ARATH      QFPELAEARQAVLVIREKL--------DSSIASFRKKLAIR-------------NLEFLQ
                                                    :

MSH1_ARATH      SIWAGTAGEDQ---IKQLKPALDSKGKKVGEEWFTTPKVEIALVRYHEASENAKARVLEL
MSH4_ARATH      ----FFFRIPQKEVQGKLPNKFTQVVKHGKNIHCSSLELASLNVRNKSAAGECFIRTETC
MSH5_ARATH      CIFGEKLDETALNRLTEFEFAFSDMDGETQRFFYHTSKTRELDNLL----GDIYHKILDM
MSH6_ARATH      VGKDEYLLEVPESLSGSVPHDYELCSSKKGVSRYWTPTIKKLLKEL----SQAKSEKESA
MSH7_ARATH      --LELFLSQFEAAIDSDFPN-------------YQNQD------------------VTDE
MSH2_ARATH      VF--RITKKEEPKIRKKLTTQFIVLETRKDGVKFTNTKLKKLGDQY----QSVVDDYRSC
MSH3_ARATH      VS--GITHLIELPVDSKVPMNWVKVNSTKKTIRYHPPEIVAGLDEL----ALATEHLAIV
                                ..

MSH1_ARATH      LRELS-----------VKLQTKINVL-VFASMLLVISKAL--FSH-ACEGRRRKWVFPTL
MSH4_ARATH      LEALMDA-----------IREDISALTLLAEVLCLLDMIVNSFAHTISTKPVDRYSRPEL
MSH5_ARATH      ERAIIRD---LLSHTLLFSAHLLKAVNFVAELDCILSLAC------V--AHQNNYVRPVL
MSH6_ARATH      LKSISQR---LIGRFCEHQEKWRQLVSATAELDVLISLAF------ASDSYEGVRCRPVI
MSH7_ARATH      NAETLTI---LIELFIERATQWSEVIHTISCLDVLRSFAI------AASLSAGSMARPVI
MSH2_ARATH      QKELVDRVVETVTSFSEVFEDLAG---LLSEMDVLLSFAD------LAASCPTPYCRPEI
MSH3_ARATH      NRASWDS---FLKSFSRYYTDFKAAVQALAALDCLHSLST------L--SRNKNYVRPEF
                                             : :  : .                    * :
MSH1_ARATH      VGFSLDEGAKPLDGASRMKLTGLSPYWFDV---SSGTAVHNT-----------VDMQSLF
MSH4_ARATH      TDSGP------------LAIDAGRHPILESI---HNDFVSNSIFMS--------EATNML
MSH5_ARATH      TVESL------------LDIRNGRHVLQE---MAVDTFIPNDTEIN--------DNGRIH
MSH6_ARATH      SGSTSD-------GVPHLSATGLGHPVLRGDSLGRGSFVPNNVKIG------GAEKASFI
MSH7_ARATH      FPESEATDQNQKTKGPILKIQGLWHPFAVAA--DGQLPVPNDILLGEARRSSGSIHPRSL
MSH2_ARATH      TS-SDAGD---------IVLEGSRHPCVEAQ--DWVNFIPNDCRLM-------RGKSWFQ
MSH3_ARATH      VDDCEPVE---------INIQSGRHPVLETI--LQDNFVPNDTILH-------AEGEYCQ
                                 :                    : *

MSH1_ARATH      LLTGPNGGGKSSLLRSICAAALLGISGLMVPAESACIPHFDSIMLHMKSYDSPVDGKSSF
MSH4_ARATH      VVMGPNMSGKSTYLQQVCLVVILAQIGCYVPARFATIRVVDRIFTRMGTMDNLESNSSTF
MSH5_ARATH      IITGPNYSGKSIYVKQVALIVFLSHIGSFVPADAATVGLTDRIFCAMGSK-FMTAEQSTF
MSH6_ARATH      LLTGPNMGGKSTLLRQVCLAVILAQIGADVPAETFEVSPVDKICVRMGAKDHIMAGQSTF
MSH7_ARATH      LLTGPNMGGKSTLLRATCLAVIFAQLGCYVPCESCEISLVDTIFTRLGASDRIMTGESTF
MSH2_ARATH      IVTGPNMGGKSTFIRQVGVIVLMAQVGSFVPCDKASISIRDCIFARVGAGDCQLRGVSTF
MSH3_ARATH      IITGPNMGGKSCYIRQVALISIMAQVGSFVPASFAKLHVLDGVFTRMGASDSIQHGRSTF
                :: *** .***  ::      ::.  *  **.    :   * :   : :        *:*

MSH1_ARATH      QVEMSEIRSIVSQATSRSLVLIDEICRGTETAKGTCIAGSVVESLDT-SGC-LGIVSTHL
MSH4_ARATH      MTEMRETAFIMQNVTNRSLIVMDELGRATSSSDGLAMAWSCCEYLLSLKA--YTVFATHM
MSH5_ARATH      MIDLHQVGMMLRQATSRSLCLLDEFGKGTLTEDGIGLLGGTISHFATCAEPPRVVVCTHL
MSH6_ARATH      LTELSETAVMLTSATRNSLVVLDELGRGTATSDGQAIAESVLEHFIEKVQC-RGFFSTHY
MSH7_ARATH      LVECTETASVLQNATQDSLVILDELGRGTSTFDGYAIAYSVFRHLVEKVQC-RMLFATHY
MSH2_ARATH      MQEMLETASILKGASDKSLIIIDELGRGTSTYDGFGLAWAICEHLVQVKRA-PTLFATHF
MSH3_ARATH      LEELSEASHIIRTCSSRSLVILDELGRGTSTHDGVAIAYATLQHLLAEKRC-LVLFVTHY
                  :  :   ::   :  ** ::**: :.* : .*  :  .    :         .. **
```

```
MSH1_ARATH      HGIFSLPLTAKNITYKAMGAENVE---------------GQTKPTWKLTDGV-CRESLAF
MSH4_ARATH      DSLAELATIYPNVKVLHFYVD---------------IRDNRLDFKFQLRDGTLHVPHYGL
MSH5_ARATH      TELLNESCLPVS-----EKIKFYTMS--VLRPDTESANMEEIVFLYRLIPGQ-TLLSYGL
MSH6_ARATH      HRLSVDYQTNPKVSLCHMACQIGEG----------IGGVEEVTFLYRLTPGA-CPKSYGV
MSH7_ARATH      HPLTKEFASHPRVTSKHMACAFKSRS-----DYQPRGCDQDLVFLYRLTEGA-CPESYGL
MSH2_ARATH      HELTALAQANSEVSGNTVGVANFHVSA------HIDTESRKLTMLYKVEPGA-CDQSFGI
MSH3_ARATH      PEIAEISNGFPGS------VGTYHVSYLTLQKDKGSYDHDDVTYLYKLVRGL-CSRSFGF
                  :                                          :::  *       ..

MSH1_ARATH      ETAKREGVPESVIQRAEALYLSVYAKDASAEVVKPDQIITSSNNDQQIQKPVSSERS---
MSH4_ARATH      LLAEVAGLPSTVIDTARIITKRITDKENKRIE-LNC----------------GKHHEIH-
MSH5_ARATH      HCALLAGVPEEVVKRAAIVLDAFESNNNVDKL-SLDKI--SSQD-QAFKDAVDKFAELDI
MSH6_ARATH      NVARLAGLPDYVLQRAVIKSQEFEALYGKNHR-K-------TDHKL------------AA
MSH7_ARATH      QVALMAGIPNQVVETASGAAQAMKRSIGENFK-S-------SELRSEF----------SS
MSH2_ARATH      HVAEFANFPESVVALAREKAAELEDFSPSSMI-INNEE--SGKRKSREDD----PDEVSR
MSH3_ARATH      KVAQLAQIPPSCIRRAISMAAKLEAEVRA------------RERNTRMGEP--EGHEEPR
                  *    .*   :  *      .

MSH1_ARATH      LEKDLAKAIVKICGKKMIEPEAIECLSIGARELPPPS-----------------TVGSSC
MSH4_ARATH      ----------RIY----RVAQRLICLKYSRQTEDSIR------QALQNLNESFTEER---
MSH5_ARATH      SKGDIHAFFQDIF----TS-----------------------------------------
MSH6_ARATH      MIKQIISS----------VA-----------SDSDYSASKDSLCELHSMANTFLRLTN--
MSH7_ARATH      LHEDWLKSLVGIS----RVAHNN-----APIGEDDY---DTLFCLWHEIKSSYCVPK---
MSH2_ARATH      GAERAHKFLKE-F-----AAIPL-----DKMELKDSL------QRVREMKDELEKDAADC
MSH3_ARATH      GAEESISALGDLF-----ADLKF-----AL-SEEDPWKAFEFLKHAWKIAGKI-RLKPTC
MSH1_ARATH      VYVMRRPDKRLYIGQTDDLEGRIRAHRAKEGLQGSSFLYLMVQGKSMACQLETLLINQLH
MSH4_ARATH      ------L-----------------------------------------------------
MSH5_ARATH      ------------------------------------------------------------
MSH6_ARATH      ------------------------------------------------------------
MSH7_ARATH      ------------------------------------------------------------
MSH2_ARATH      HWLRQFL-----------------------------------------------------
MSH3_ARATH      SF----------------------------------------------------------

MSH1_ARATH      EQGYSLANLADGKHRNFGTSSSLSTSDVVSIL
MSH4_ARATH      --------------------------------
MSH5_ARATH      --------------------------------
MSH6_ARATH      --------------------------------
MSH7_ARATH      --------------------------------
MSH2_ARATH      --------------------------------
MSH3_ARATH      --------------------------------
```

Figure S1. Multiple Sequence Alignment of MSH Proteins.
'*' position with a fully conserved residue; ':' groups with strong similarity (> 0.5 in PAM 250 matrix); '.' groups with weak similarity (=< 0.5 in PAM 250 matrix)

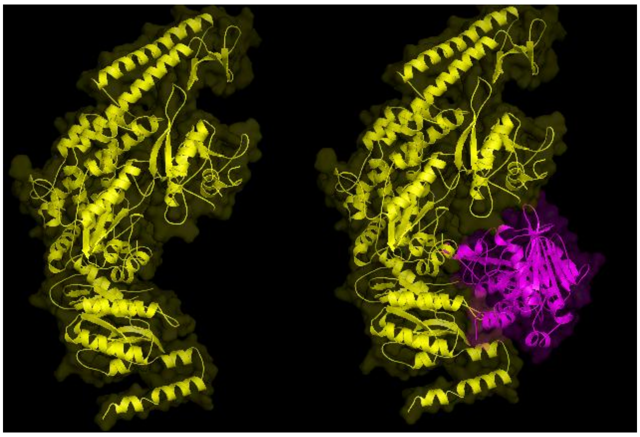

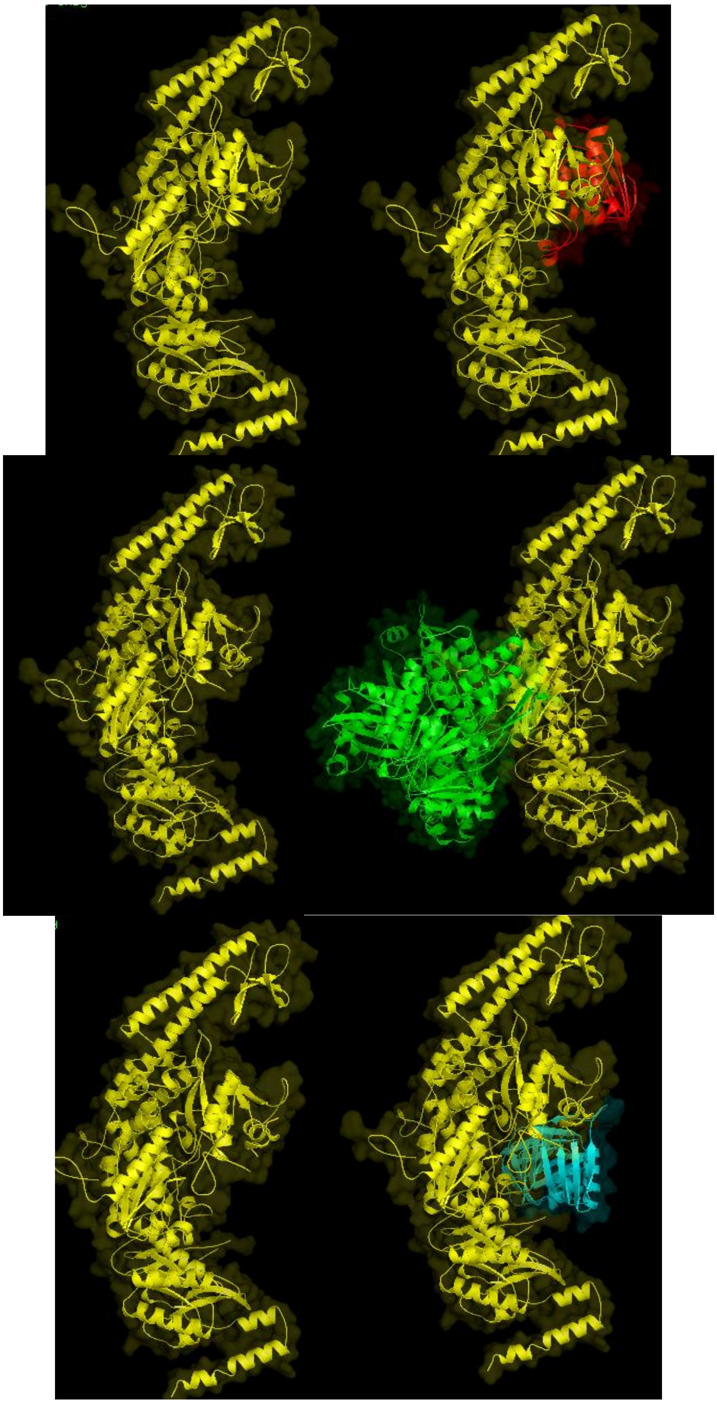

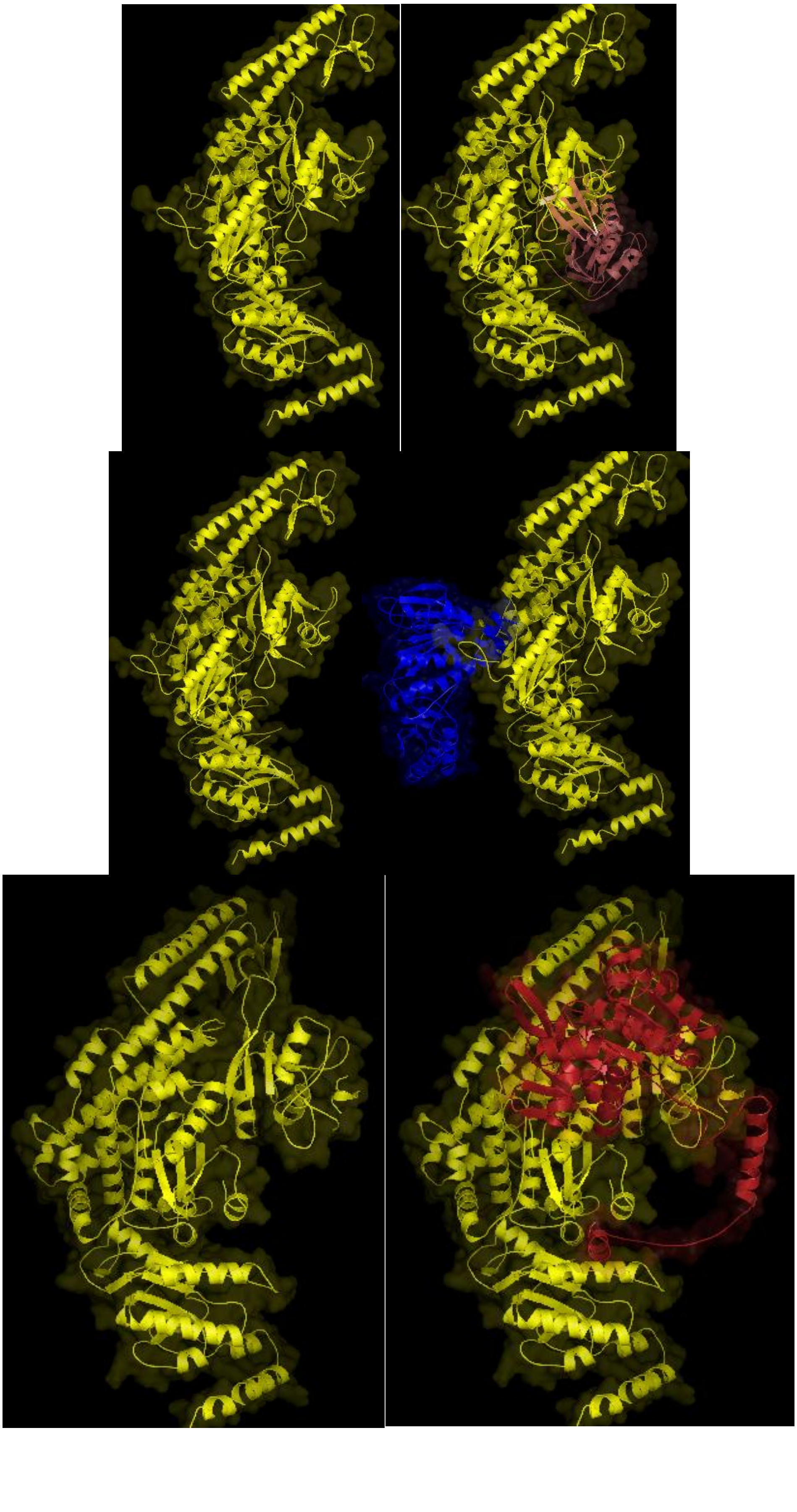

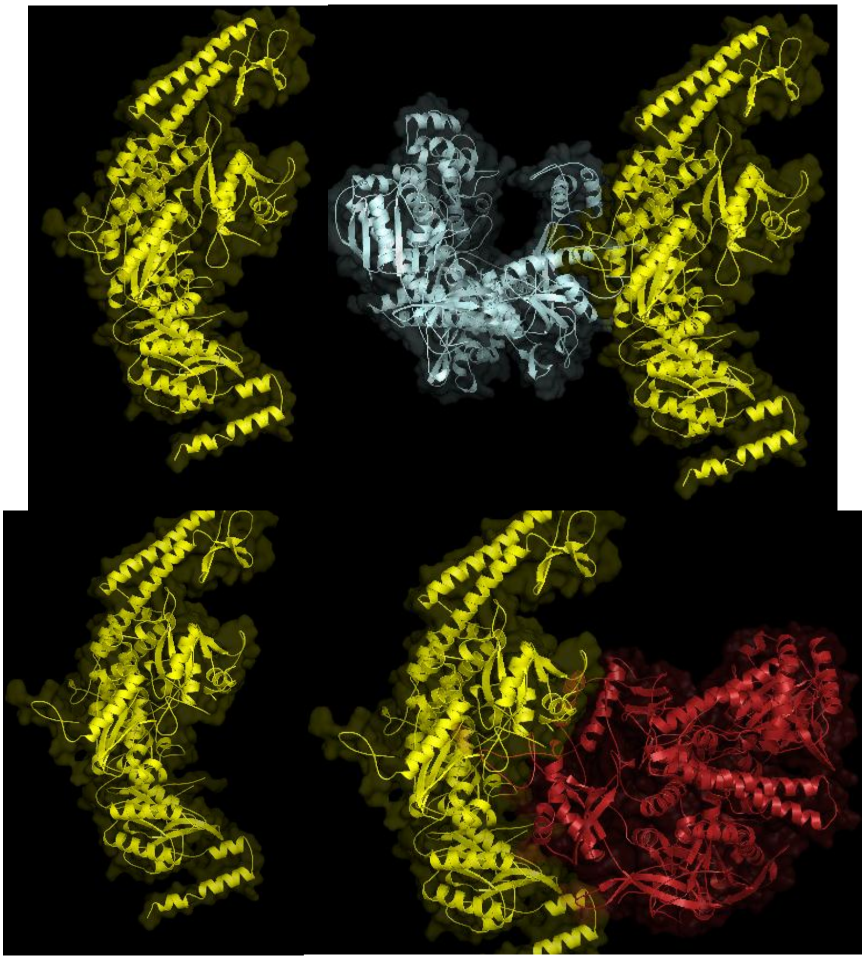

Figure S2. MSH1 interactions. Single MSH1 is shown on the left in yellow and interaction is on the right. MLH1: magenta; MLH3: red; MSH2: green; PCNA1: cyan; PCNA2: light-red; PMS1: blue; RECA3: red; TIL1: gray; TIL2: bright red

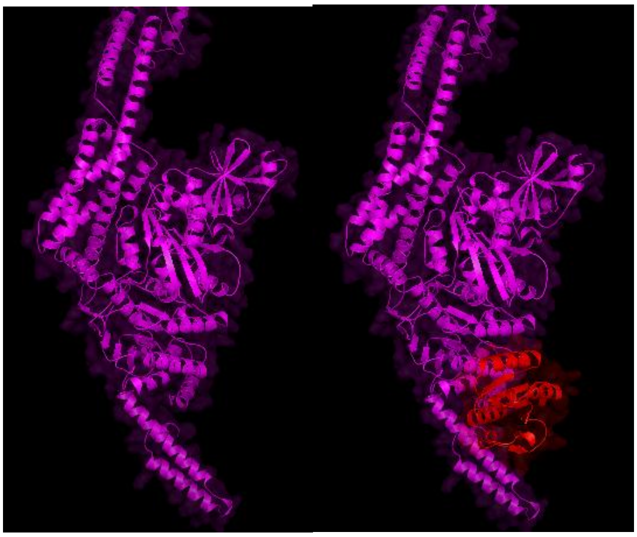

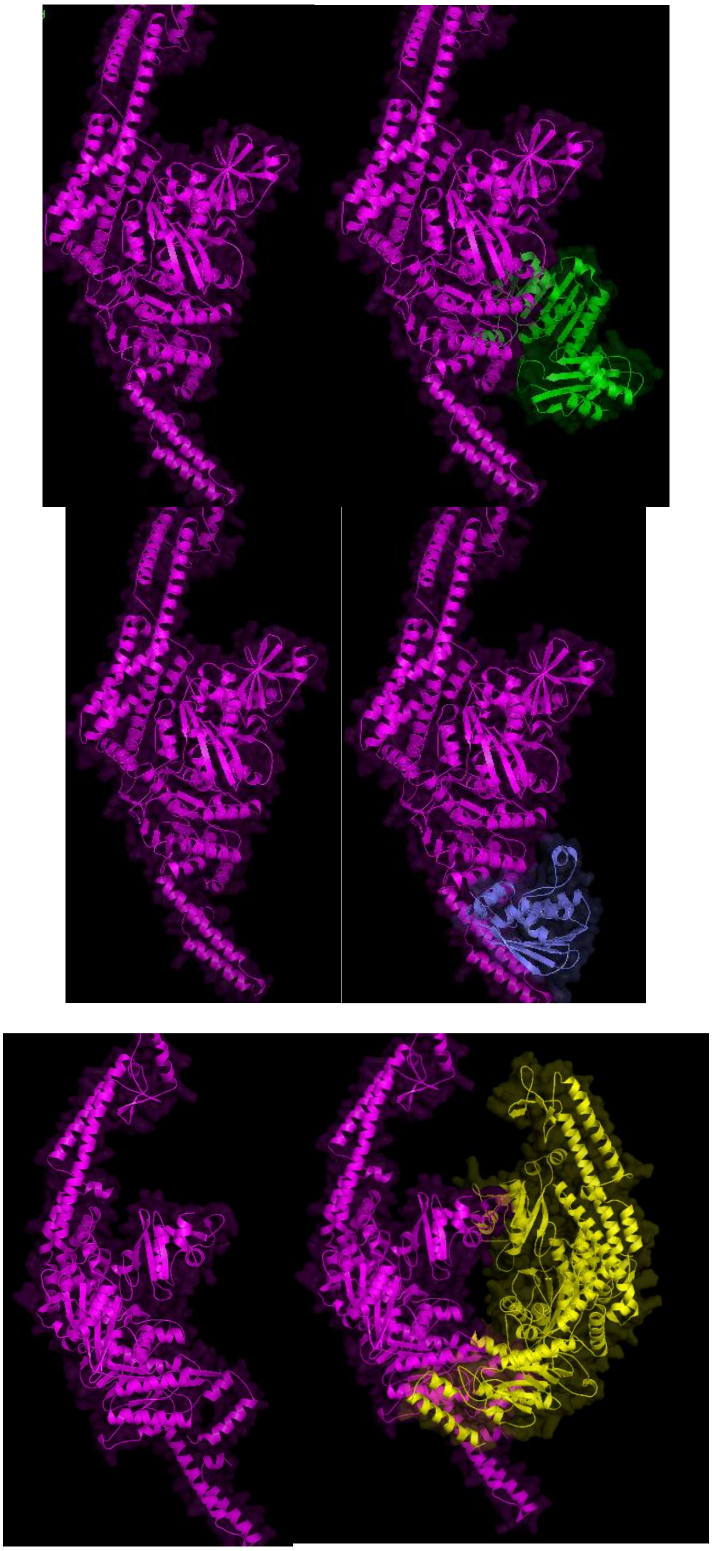

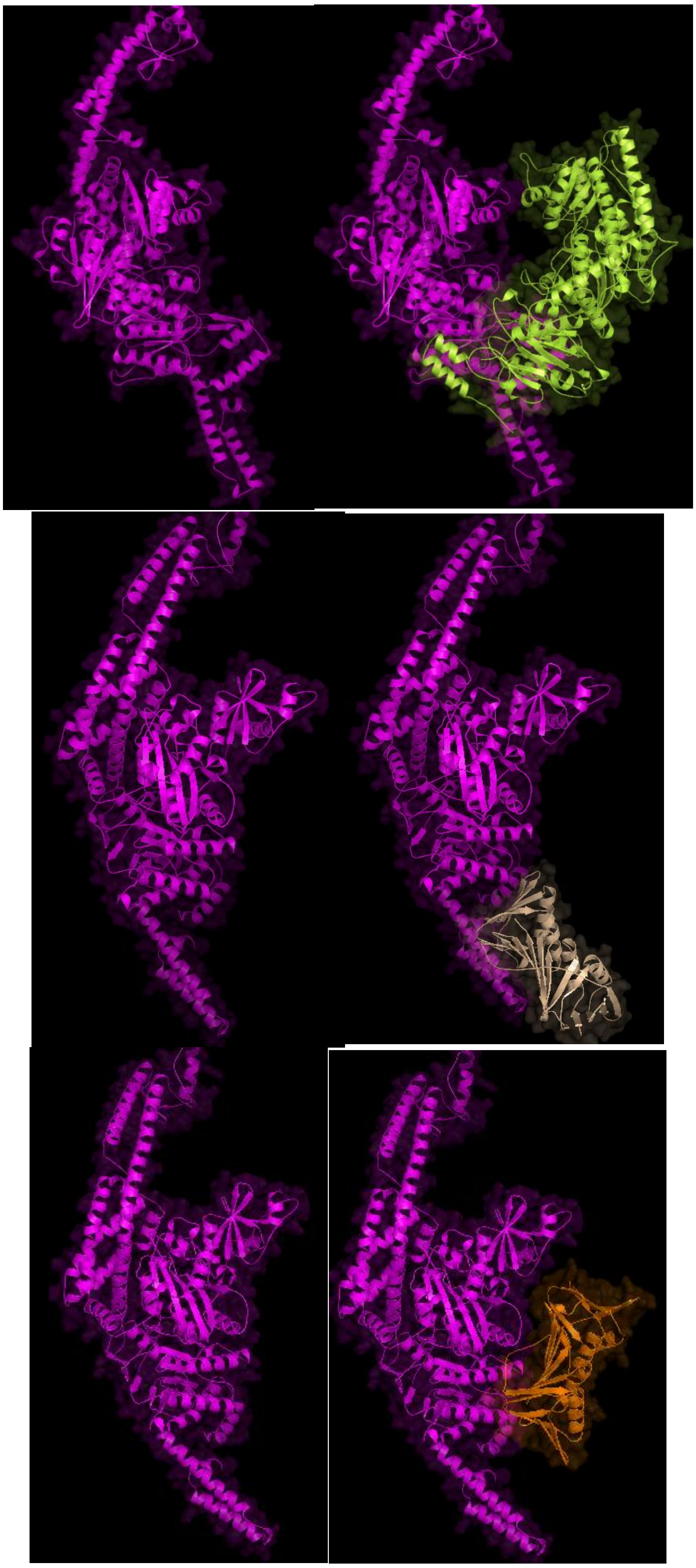

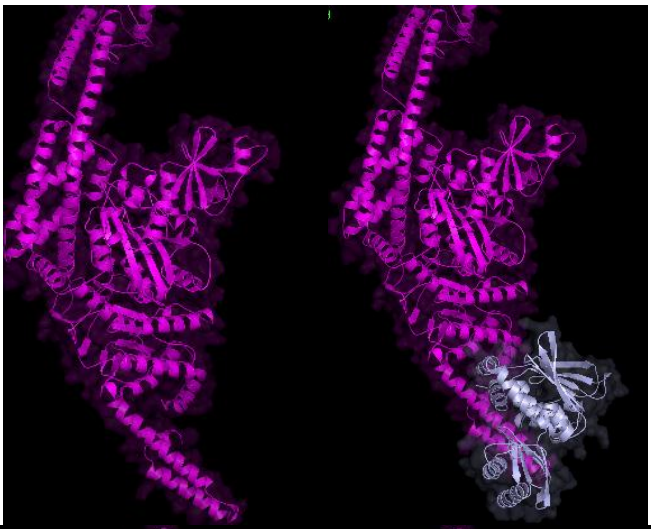

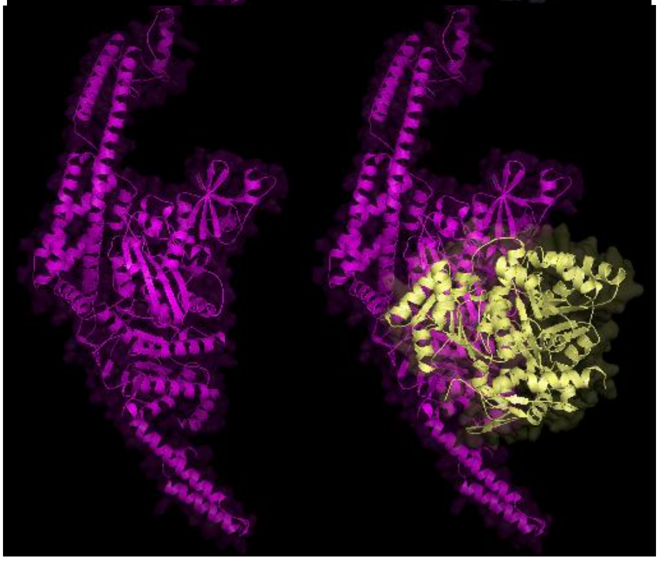

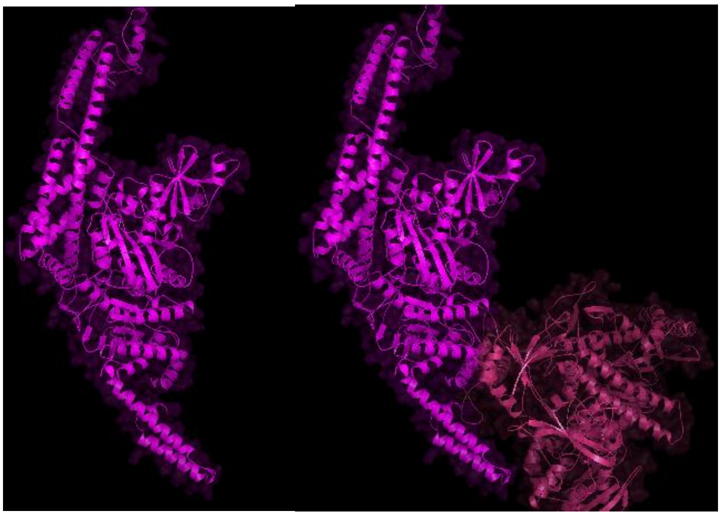

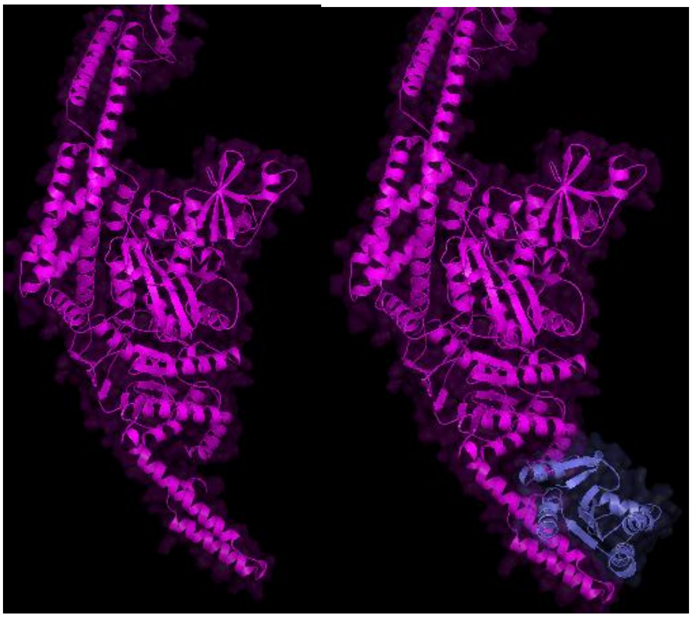

Figure S3. MSH2 interactions. *Single MSH2 is shown on the left in purple and interaction is on the right. ERCC1: red; MLH1: green; MLH3: slate blue; MSH6:yellow; MSH7: lemon; PCNA1: beige; PCNA2:orange; PMS1: light blue; RECQSIM: white; TIL1: red; UVH1: blue.*

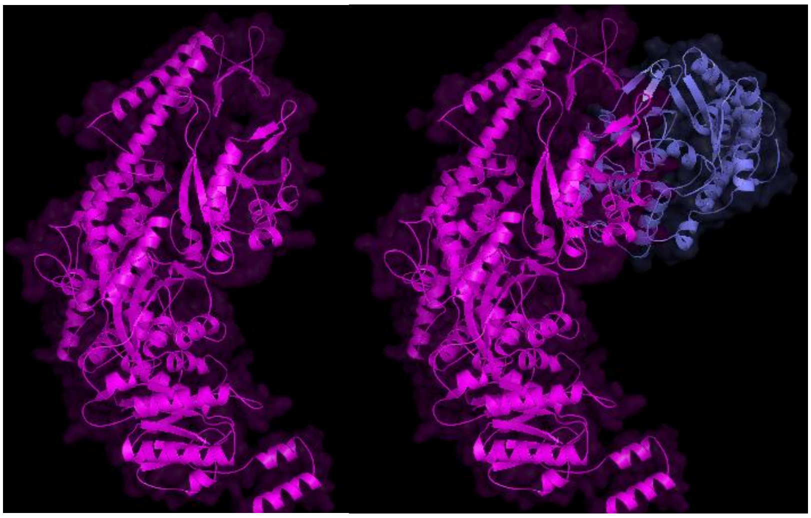

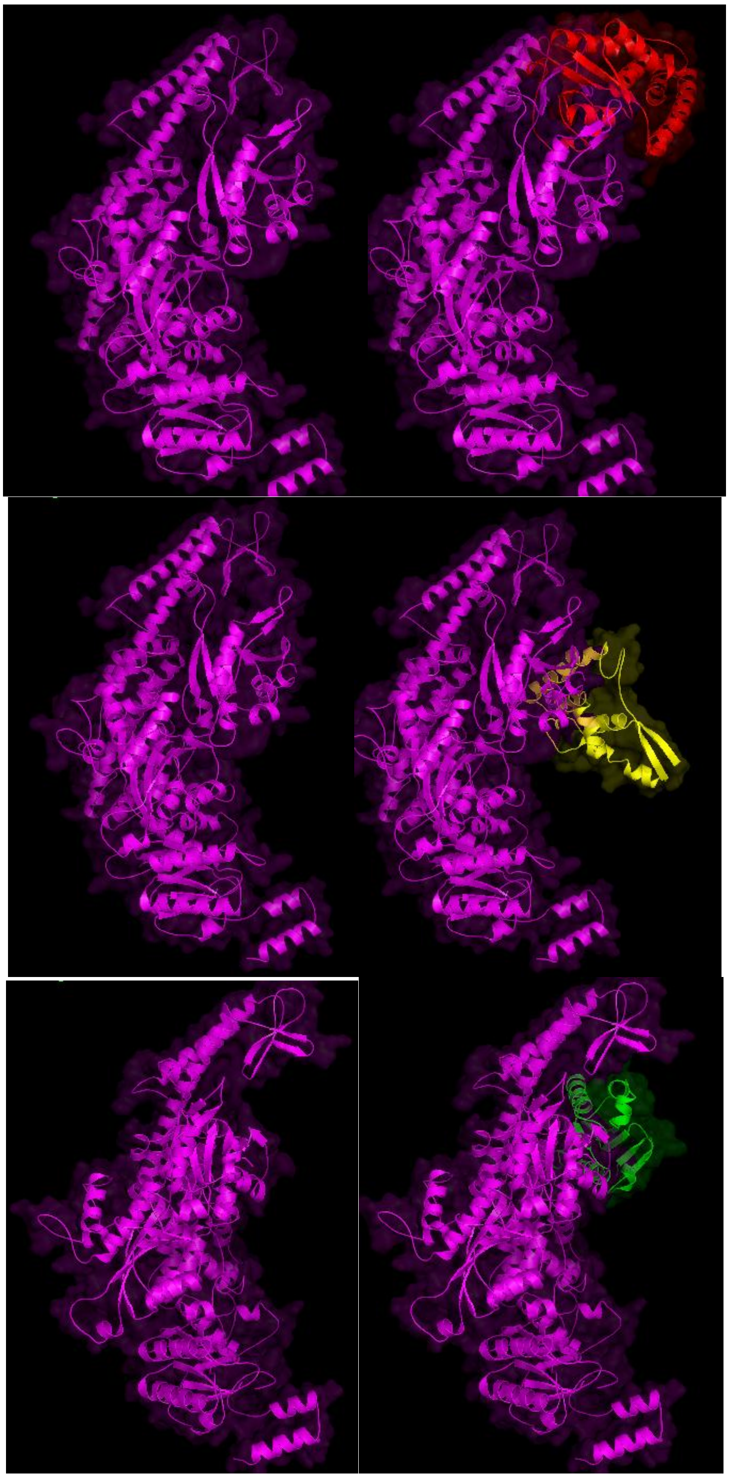

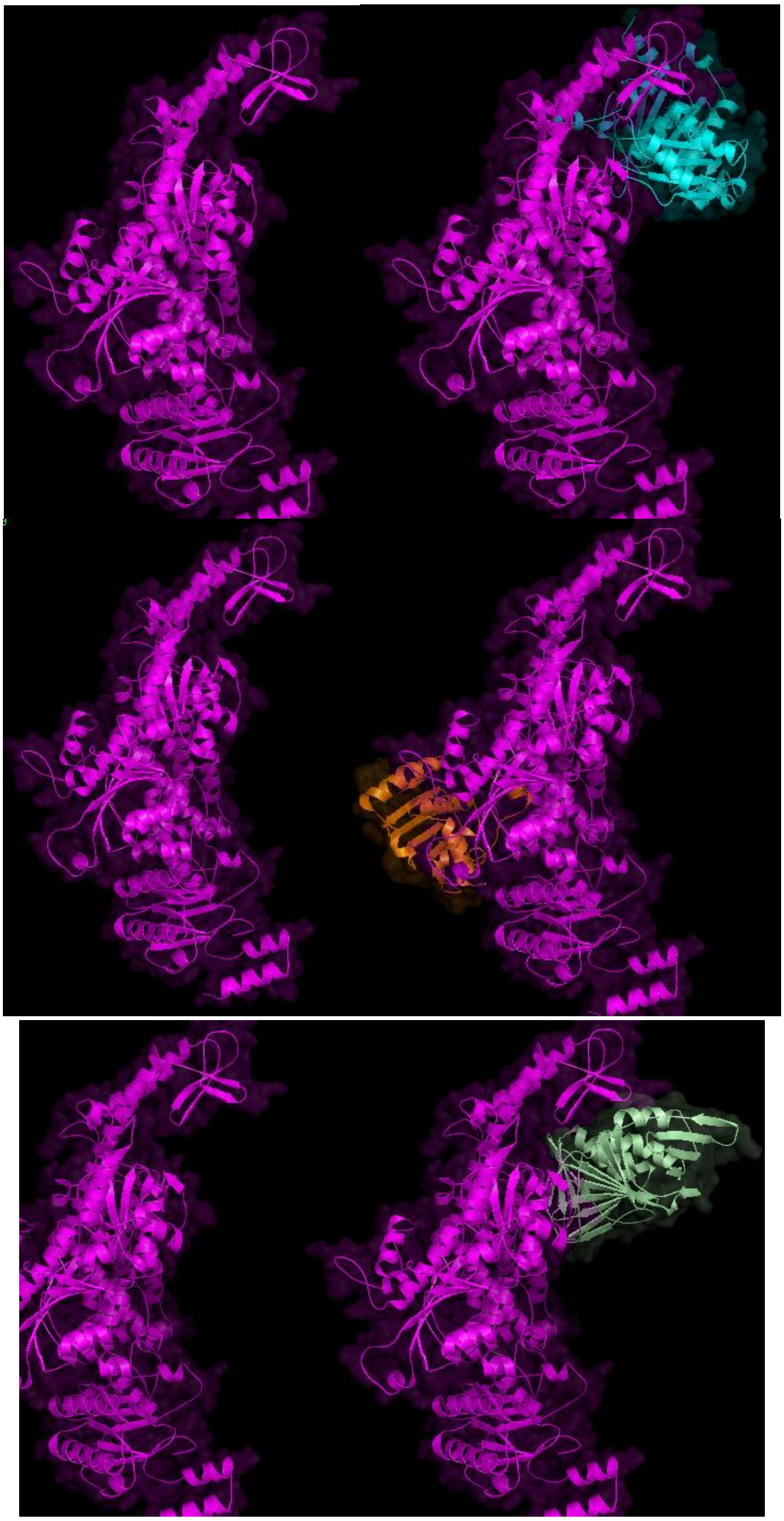

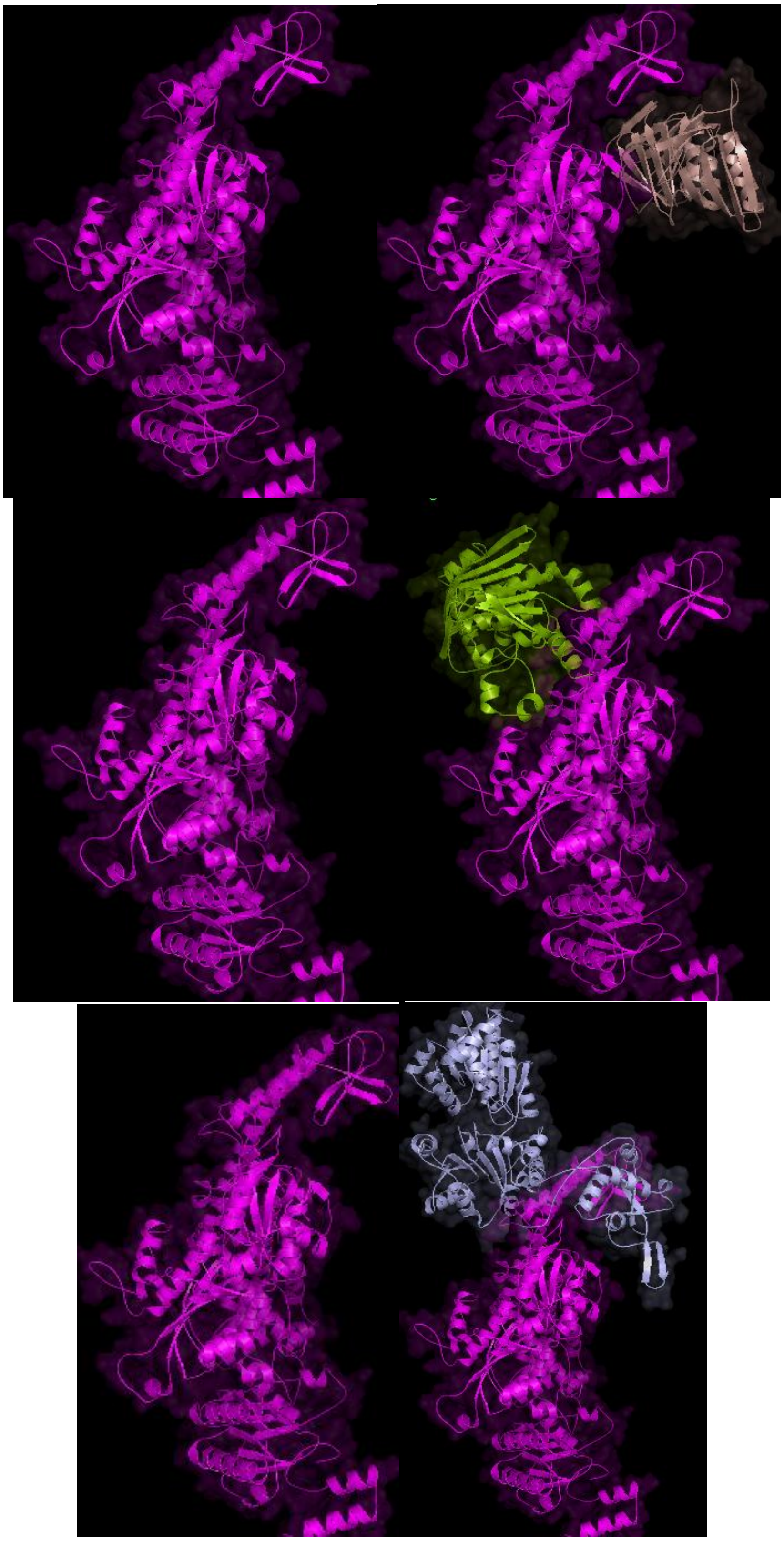

Figure 4. MSH3 interactions.*Single MSH3 is shown on the left in purple and interaction is on the right. Interactions from top to bottom: At1G18090: blue; At1G29630: red; At2G02550: yellow; ERCC1: green; MLH1:cyan; MLH3:orange; PCNA1:light green; PCNA2: beige; PMS1: yellow; RECQSIM: blue.*

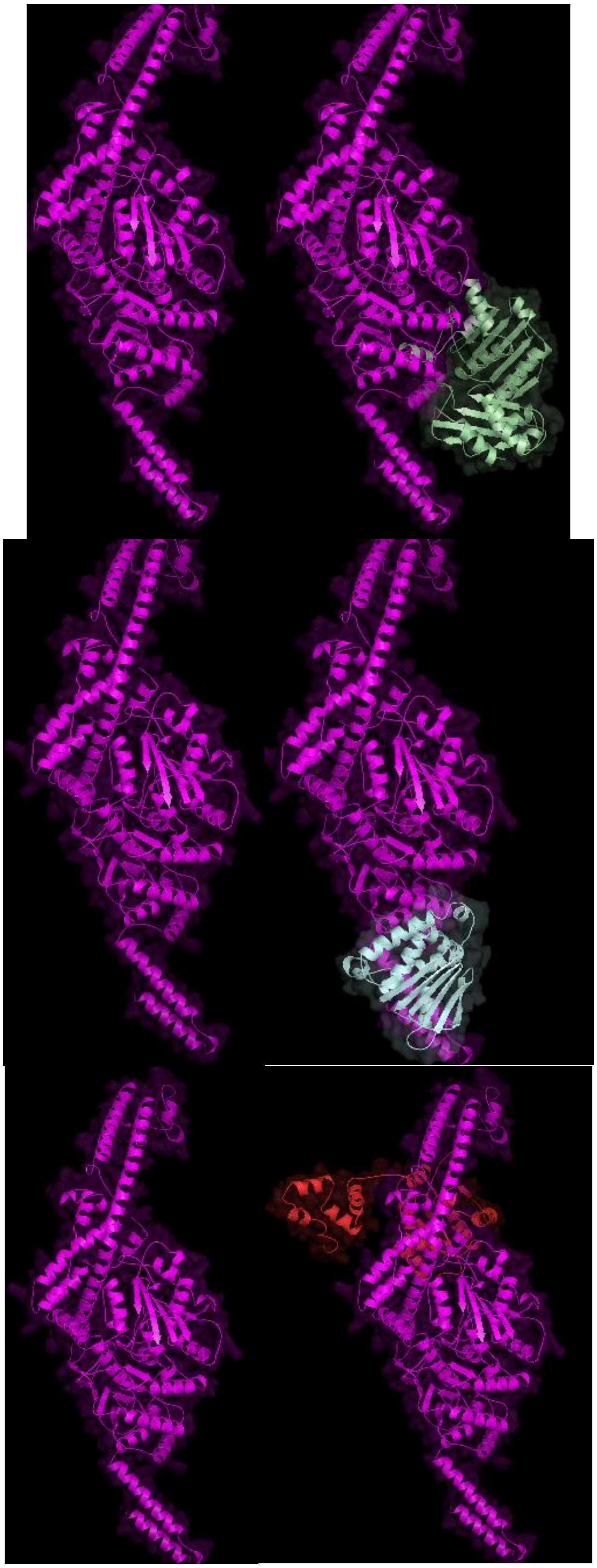

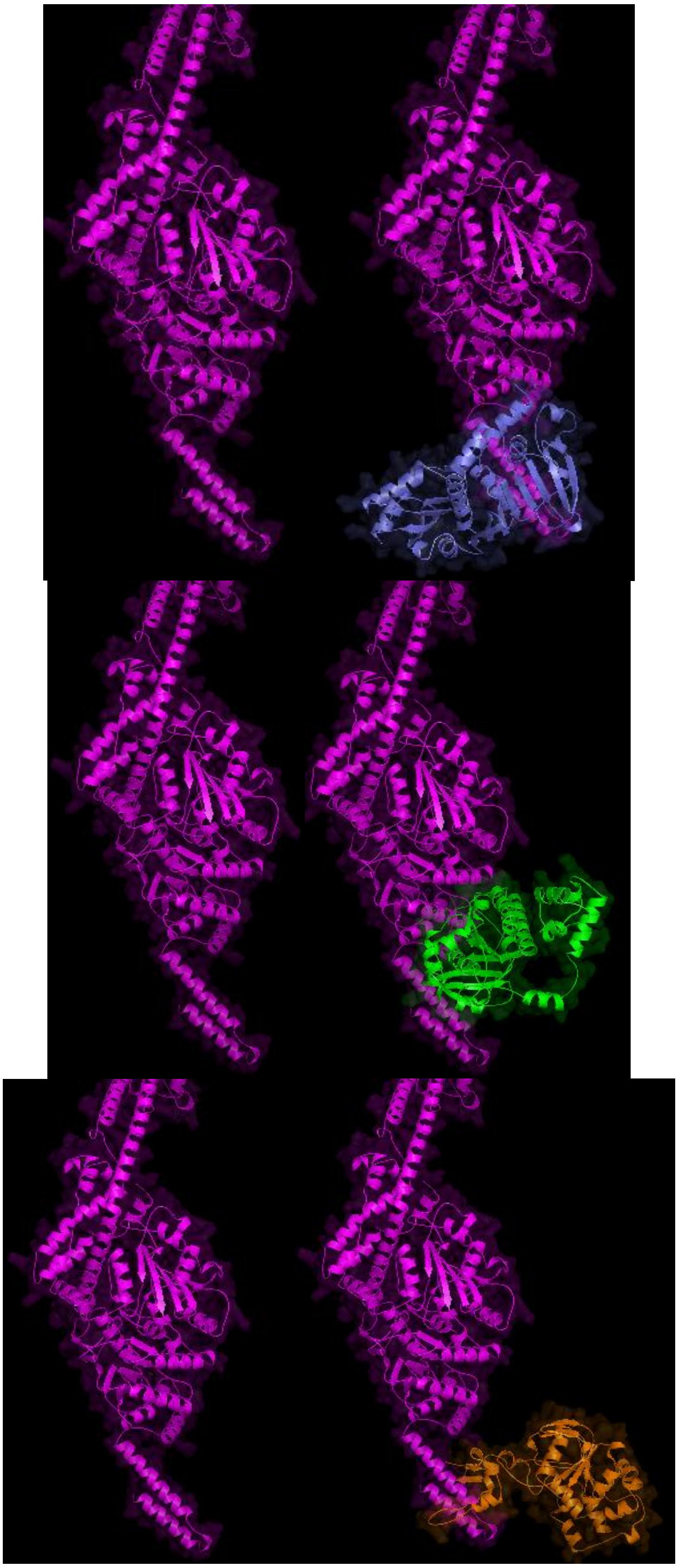

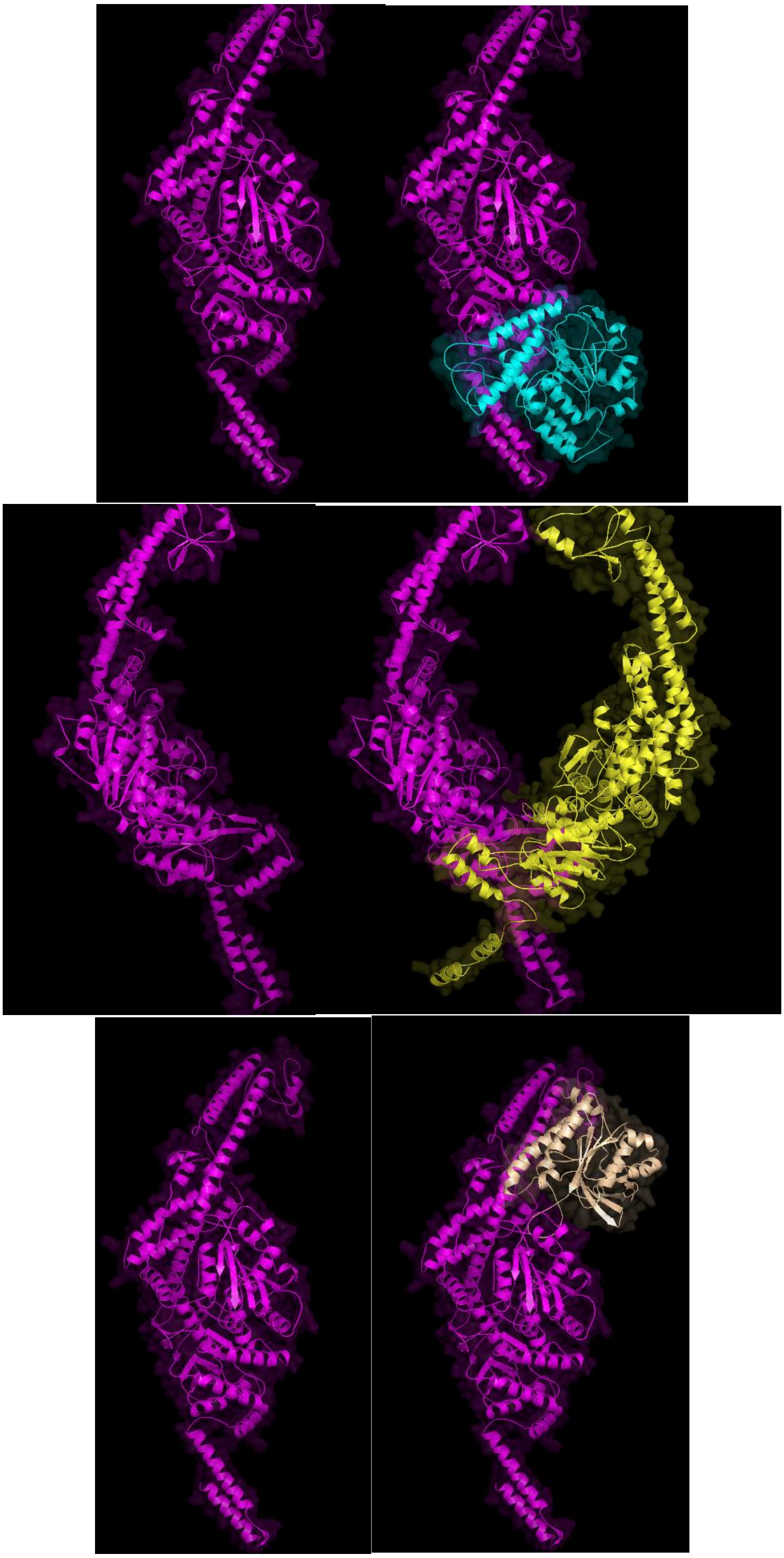

Figure S5. MSH4 interactions.Single MSH4 is shown on the left in purple and interaction is on the right. Interactions from top to bottom: MLH1: white; MLH3: light blue; MUS81:red; PMS1:blue; RAD51:green; SPO11-1:orange; SPO11-2: cyan; MSH5: yellow; DMC1: wheat .

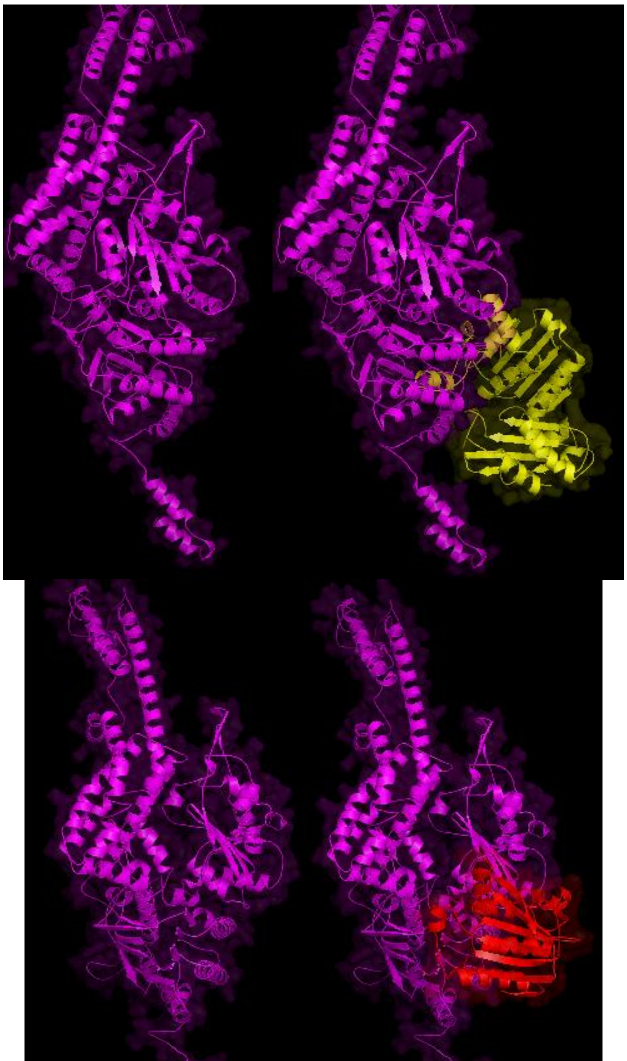

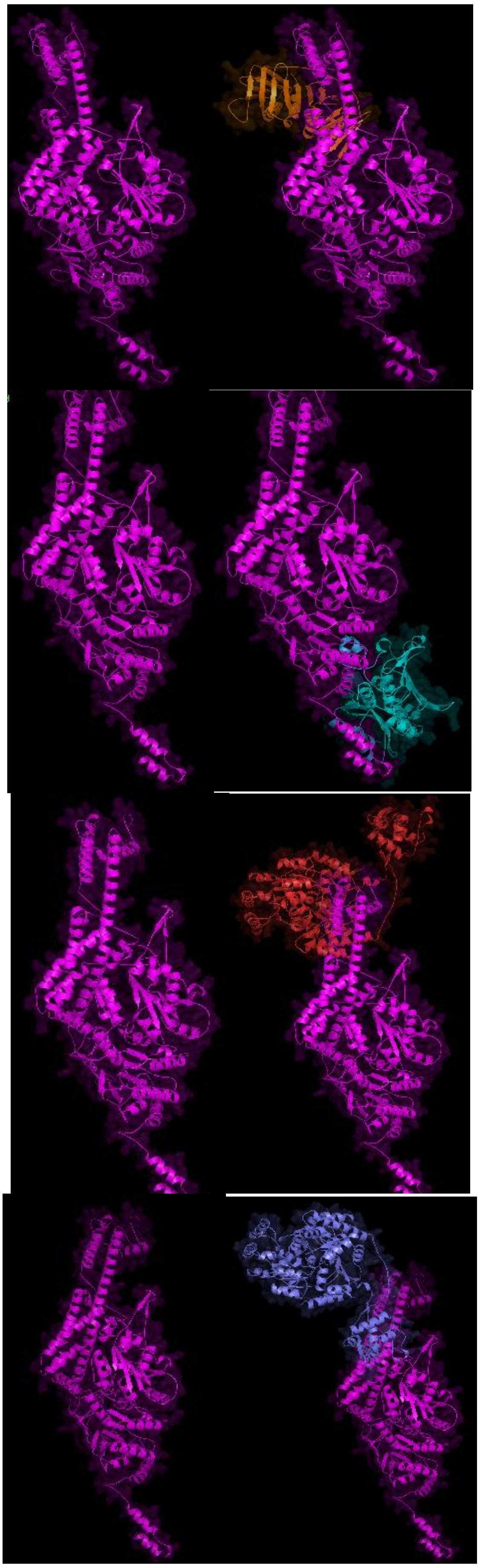

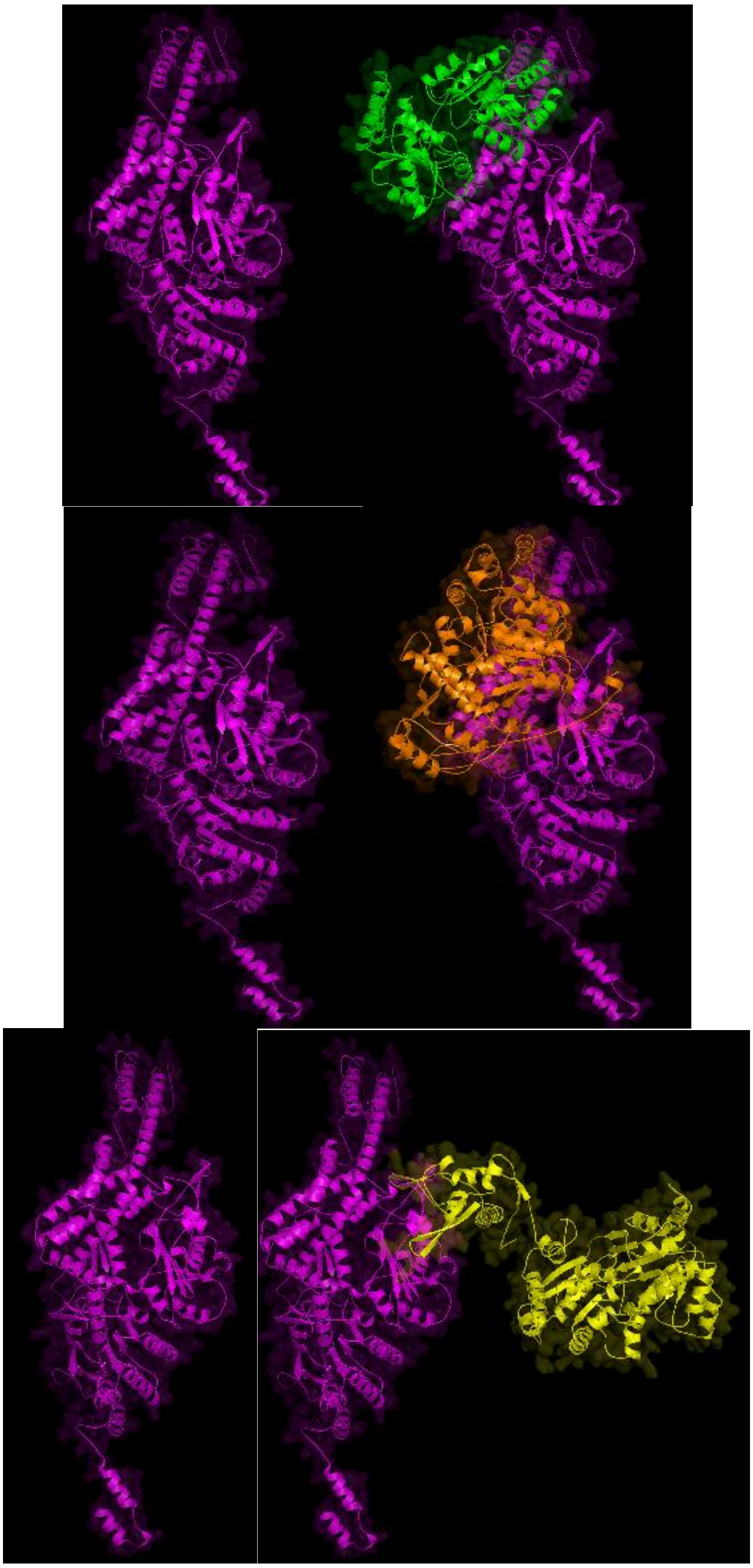

Figure S6. MSH5 interactions.Single MSH5 is shown on the left in purple and interaction is on the right. Interactions from top to bottom: MLH1: yellow; MLH3: red; PCNA1: red; PCNA2: orange; PMS: cyan; RECQ4A: red; RECQ4B: blue; RECQl1: green; RECQl3: orange; RECQSIM: yellow.

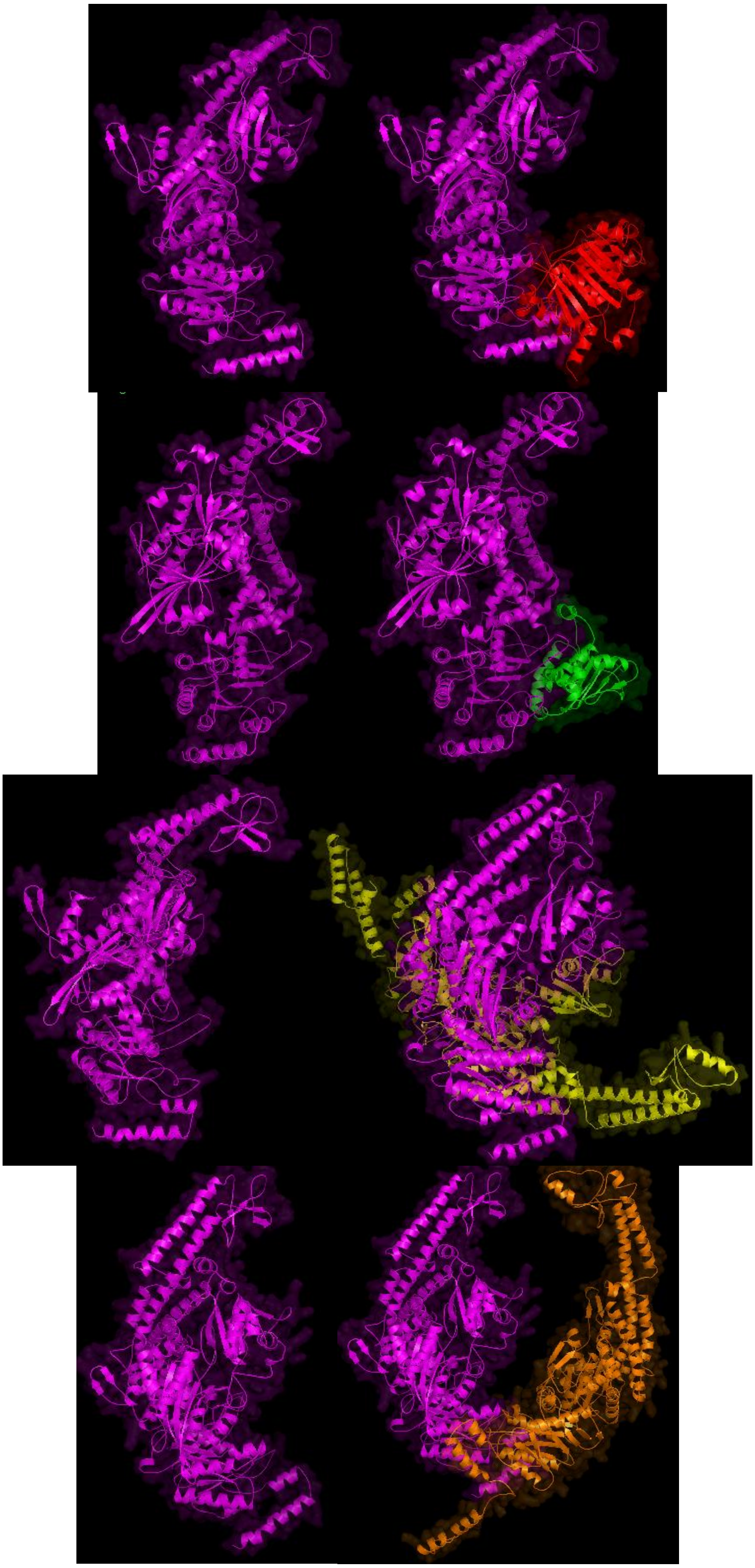

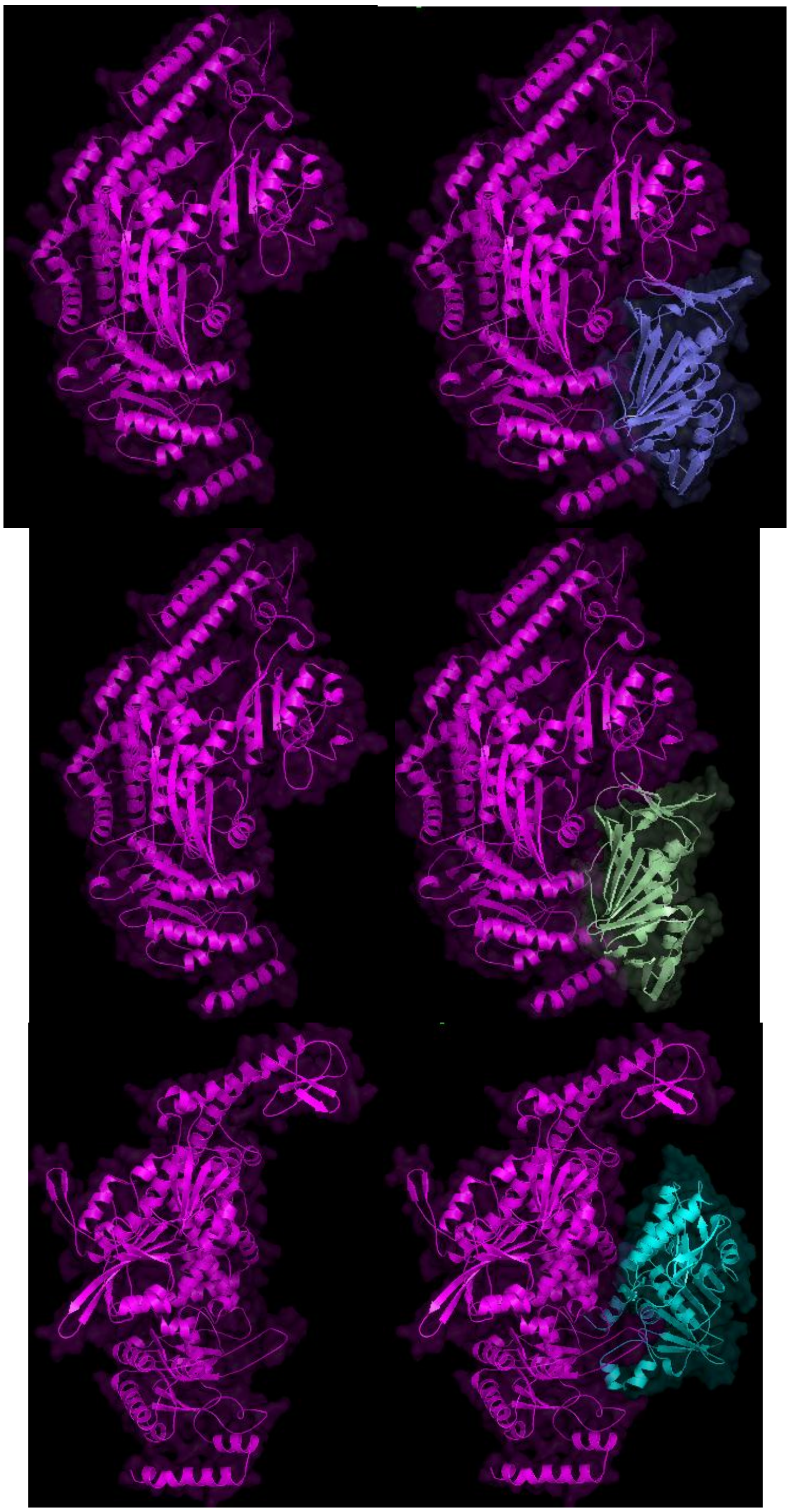

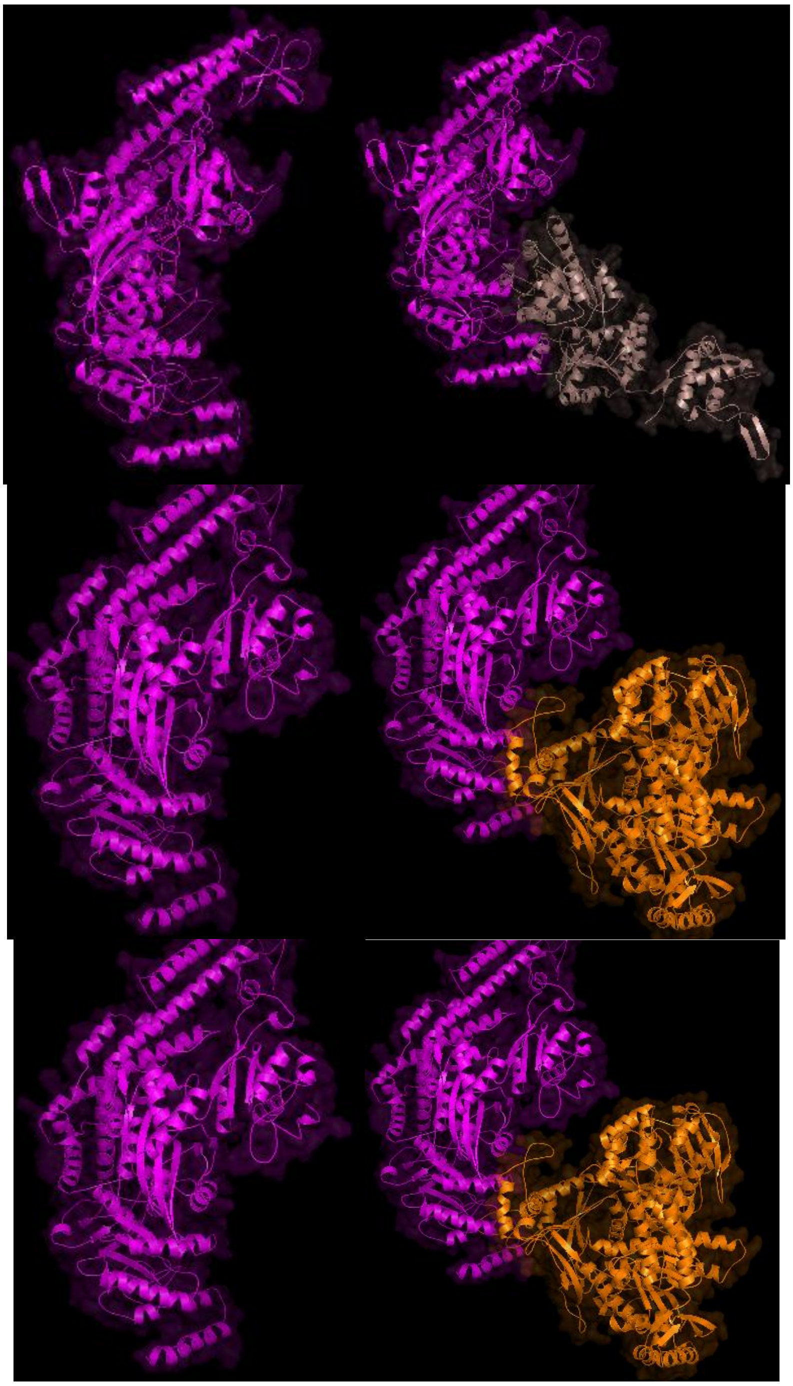

Figure S7. MSH6 interactions.Single MSH6 is shown on the left in purple and interaction is on the right. Interactions from top to bottom: MLH1: red; MLH3: green; MSH2: yellow; MSH5:orange;PCNA1: blue; PCNA2: turquoise; PMS1:cyan; RECQSIM: beige; TIL1: orange;TIL2: orange .

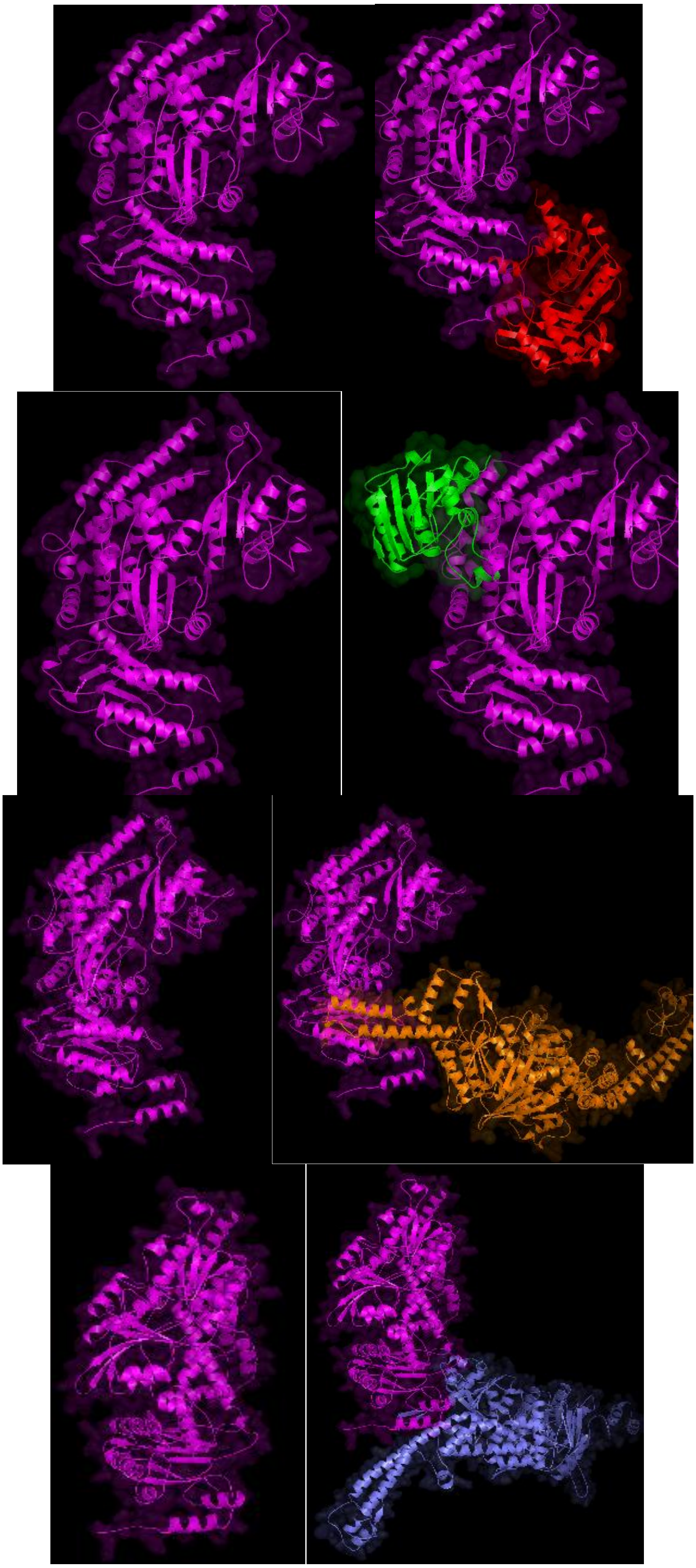

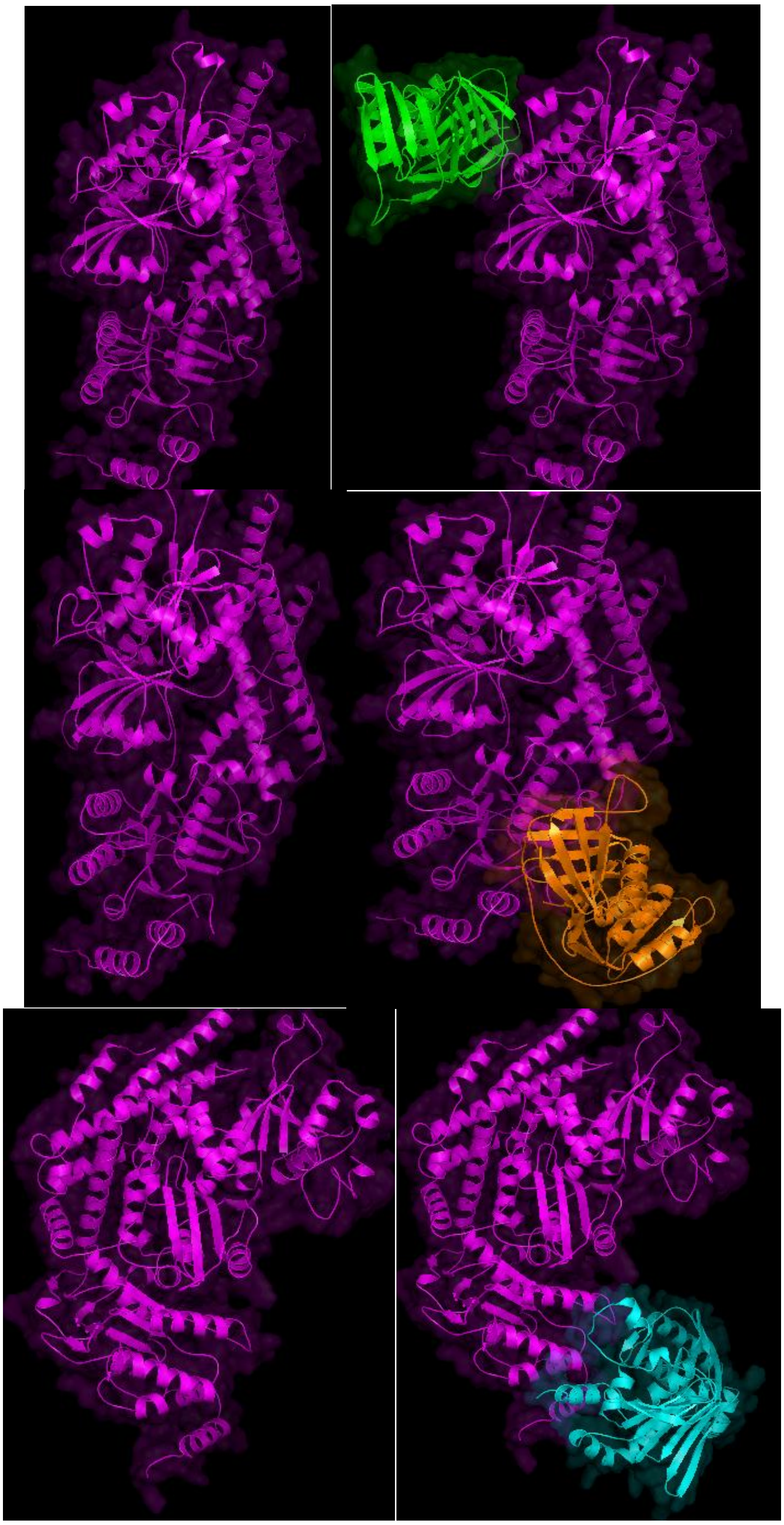

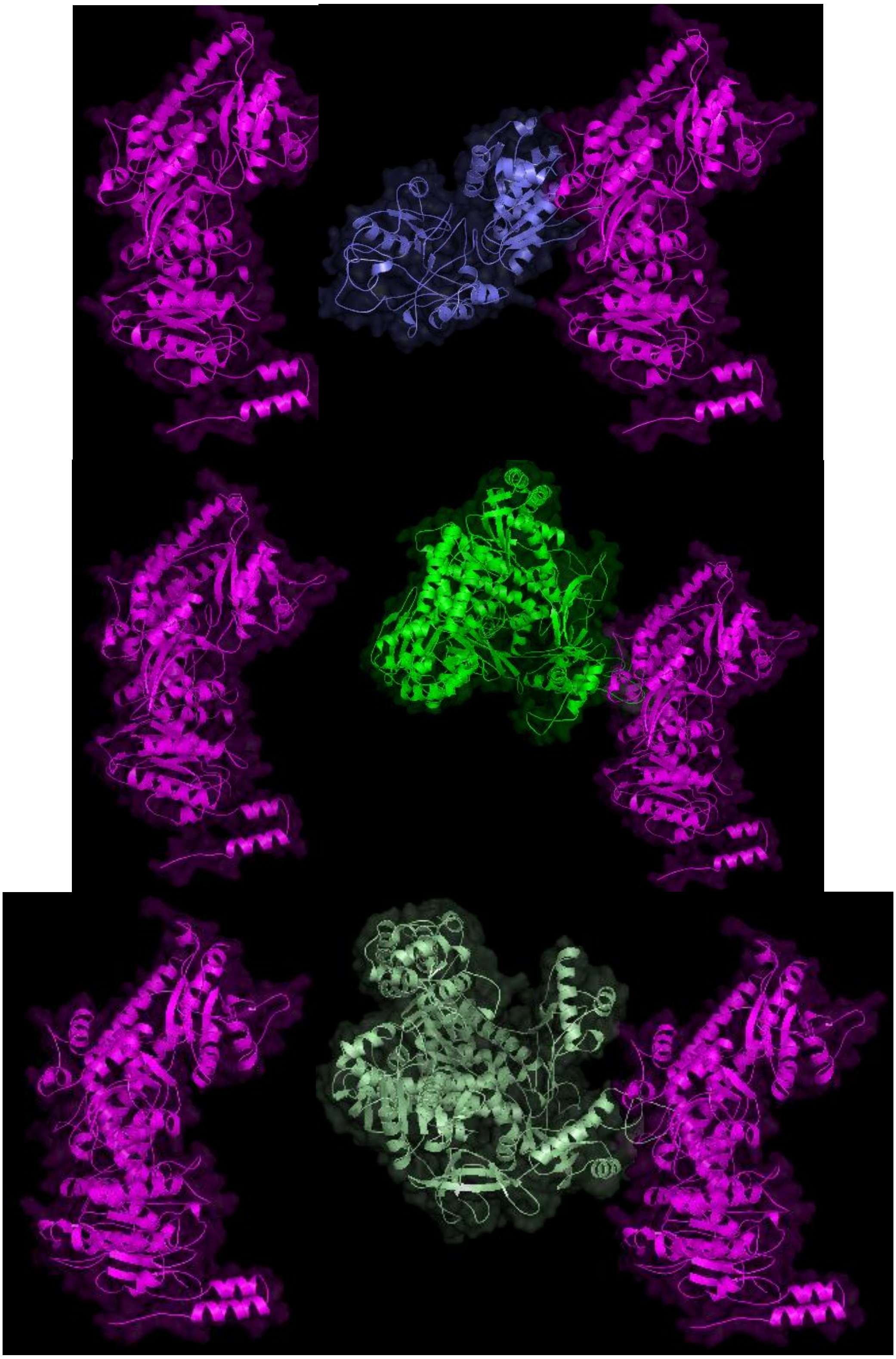

Figure S8. MSH7 interactions.Single MSH7 is shown on the left in purple and interaction is on the right. Interactions from top to bottom: MLH1: red; MLH3: green; MSH2: orange; MSH5: blue; PCNA1:green; PCNA2: orange; PMS1:cyan; RECQSIM: blue; TIL1: green; TIL2: gray .

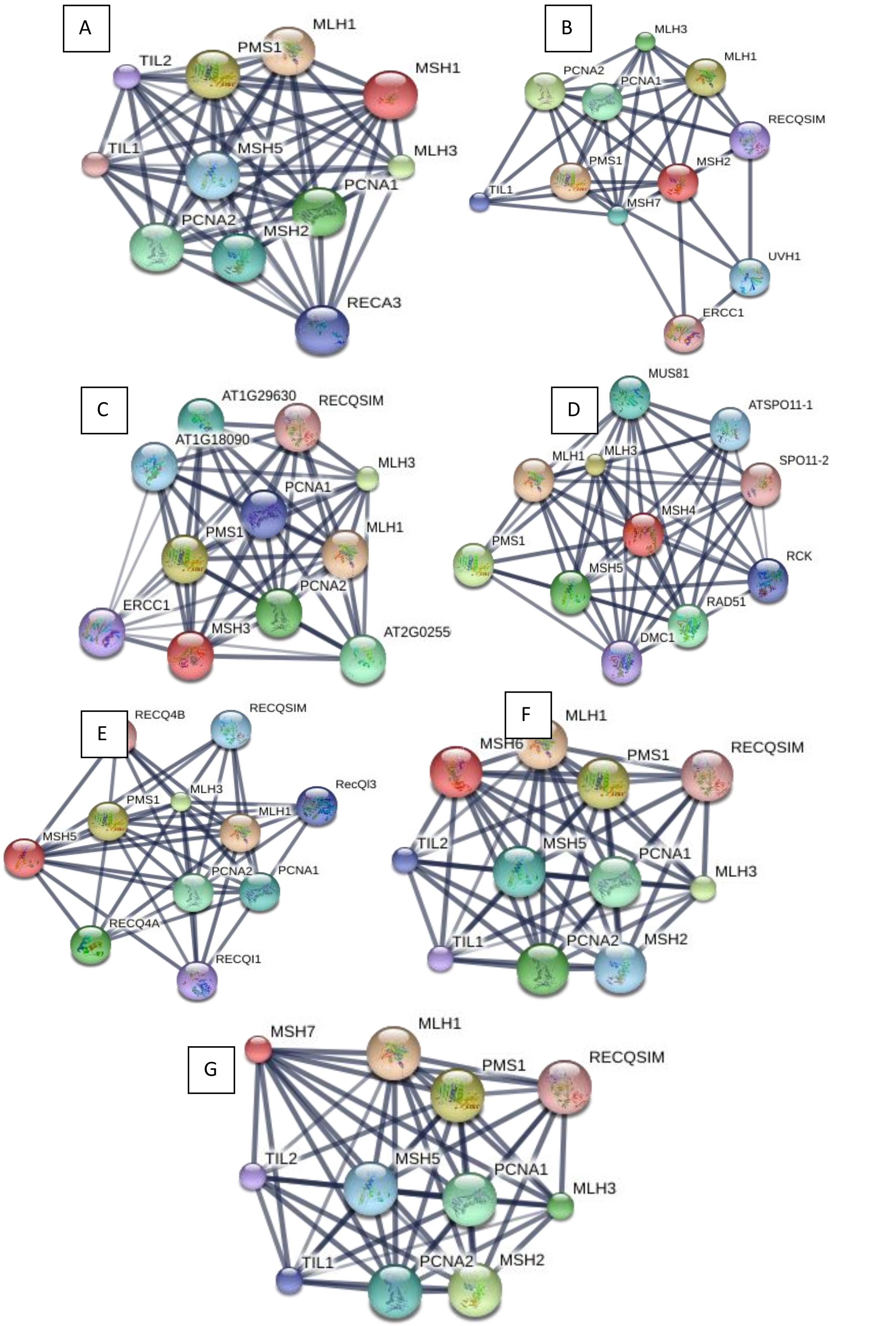

Figure S9. STRING interactome of AtMSHs. (A)MSH1;(B)MSH2;(C)MSH3;(D)MSH4;(E)MSH5;(F)MSH6;(G)MSH7.

Table S1. Docking sitesof protein-protein interactions; MSH homologs and their interactome.

Chain	Interacting chain	Residues
A (MSH1)	X (MLH1)	T67 L72 T73 P93 S94 Y104 D111 C156 E160 Y161 V282 R286 R289 C290 T306 C307 W309 L370 T374 Q375 E382 G383 I384 P385 C386 A733 S734 R735 R779 K812 S813 Y814 D815 P817 V818 D819 G820 K821 S822 S823 Q825 V826 K910 E932
X (MLH1)	A (MSH1)	V37 N38 A41 E44 V45 H87 R91 E92 P95 R100 H101 F109 E110 D111 L112 F113 S114 L115 M118 F120 E123 K141 Q143 I144 Y147 A162 C262 T263 S294 R298 R324 H326 V327 I329 N330 V338 L340 L341 N342 Q343 E344 I345 E348 M349 S352
**Chain**	**Interacting chain**	**Residues**
A (MSH1)	X (MLH3)	E146 F147 Y161 A162 G163 L164 N165 C180 P181 M183 N184 R186 Q187 T188 D191 G235 V236 D237 D239 L240 D241 F242 P243 D244 P380 T381 E382 P385 K389 V390 P393 S394 T395 S401 R405 L445 L448 L449 A454 N455 I457 M486 T489 W490 T493 G494 I497 D498 D500 T501 N504 W508 E563 K564 S565 A566 T615 K667
X (MLH3)	A (MSH1)	L7 V11 R12 M15 R16 G18 I19 I20 F22 D23 R26 E29 E73 R74 Y75 D82 F83 T84 V86 E87 T88 A89 S90 F95 R96 Q156 P157 V158 R159 K161 Y162 Q164 S165 K169 E172 S173 K175 K176 F179 R180 L183
**Chain**	**Interacting chain**	**Residues**
A (MSH1)	X (PCNA1)	D191 R194 V236 D237 D241 F242 P243 E382 P385 C386 K389 V390 L391 P393 S394 T395 C396 S401 R405 C439 V440 S441 S442 A443 K444 L445 L448 A454 C706 E707 R709 R710 R711 K712 W713 L730 D731 G732 A733 S734 R735 A753
X (PCNA1)	A (MSH1)	D21 L22 N27 S32 T33 Q38 D41 S42 S43 H44 V45 L51 R53 D120 D122 E124 H125 L126 G127 I128 P129 D130 H134 K164 K200 E201 P202 L205 S206 A208 R210 S231 E232 L233 P234 Y246 R248 A252 P253
**Chain**	**Interacting chain**	**Residues**
A (MSH1)	X (PCNA2)	R186 Q187 D191 R194 N195 V236 D237 D239 L240 D241 F242 P243 P380 E382 P385 C386 K389 V390 L391 P393 S394 T395 C396 S397 G398 S401 R405 C439 V440 S441 S442 A443 L448 A454 N455 Y456 I457 E458 D498 C706 E707 R710 K712 L730 D731 A733 S734 R735 T752 A753 V905 K910
X (PCNA2)	A (MSH1)	D25 A39 M40 H44 V45 D120 D122 S123 E124 L126 G127 I128 P129 D130 A131 E132 H134 I136 R138 V159 S161 T163 K164 E165 K168 T177 N179 V181 R183 N200 E201 P202 S204 L205 S206 S231 E232 L233 P234 R248 A252 P253
**Chain**	**Interacting chain**	**Residues**
A (MSH1)	X (PMS1)	E311 F312 E314 G315 G316 L317 L318 W319 G320 E321 C322 R325 N326 L335 L339 L348 V352 F354 R355 N356 V357 N358 R364 H473 N536 E537 F539 Y540 D541 I685 N686 V689 F690 M693 L694 I697
X (PMS1)	A (MSH1)	I20 N21 R22 V24 I25 H26 R27 I28 C29 Q32 L35 K88 H89 H90 T91 L94 T104 F108 G110 E111 S114 S166 N167 P169 V170 R171 S172 K173 E174 K176 R177 I179 R180 G209 K210 T322 P323 D324 K325
**Chain**	**Interacting chain**	**Residues**
A (MSH1)	X (RECA3)	D70 V71 S94 T95 Q97 I99 E100 R101 L102 M103 T105 N106 L107 L108 R115 L119 W126 E127 Q130 F131 R134 V205 Q206 P210 R212 S213 R214 G216 F218 R253 A255 T277 E278 E279 L281 V282 H301 A303 T306 C307 G320 E321 W329 D333 L339 K343 D344 Y346 L348 D349 V352 S353 R355 H369 L370 G371 H471 M472 H473 H475 A476 E477 V479 I481 L482 N665 E672 K812 Y814 K821 S822 S823 Q825 V826 S829 E830 R832 S833 S836
X (RECA3)	A (MSH1)	E35 R37 Y38 L39 S40 S41 L42 V43 E44 A45 S46 D47 C48

		E49 L50 D51 E52 L66 A69 Q72 L73 F77 D78 S81 K82 L83 S84 L85 K91 R92 R117 C210 D213 A214 E218 R219 Y220 R221 D222 R226 T229 Q230 R233 K234 H236 Y237 G240 Y241 H255 V256 K257 S258 N259 M260 H261 F262 P263 H264 V268 N273 P276 F277 H278 Q305 K315 K317
**Chain**	**Interacting chain**	**Residues**
A (MSH1)	X (TIL1)	P245 M267 A269 S271 R288 F312 R325 N356 N358 V359 P360 S361 K362 N363 R364 P365 L399 L402 Y403 D406 L407 P412 D415 I416 K419 I420 C460 K463 I517 S518 E521 N527 K530 N536 E537 F539 Y540 D541 M542 R547 K551 N686 V689 F690 M693 L694 I697 R841
X (TIL1)	A (MSH1)	M98 E99 E101 Y105 R108 K128 Q164 A165 K166 F167 D168 A169 L170 E171 A172 Y173 E174 S175 I176 L177 A178 G179 K180 R181 Q183 R184 P185 Q186 D187 C188 L189 P497 E498 F500 N503 K652 Y655 Y656 K659 K660 E663 S664 K684 Q687
**Chain**	**Interacting chain**	**Residues**
A (MSH1)	X (TIL2)	L72 P93 S94 L96 Y104 T105 L107 G109 L110 D111 P112 S113 L114 K132 P136 R137 I153 D154 I157 E160 Y161 A162 G163 L164 N165 N184 Q187 R194 N195 G196 Y197 H224 H292 L293 F294 L295 A297 R300 R308 W309 E337 S340 R341 D344 S816 P817 V818 K821
X (TIL2)	A (MSH1)	K87 D88 N89 M90 E91 R104 Q105 A157 K158 F159 N160 A161 L162 E163 A164 Y165 E166 S167 I168 L169 Q175 R176 P177 Q178 D179 C180 L181 D182 F492 G495 K644 Y648
**Chain**	**Interacting chain**	**Residues**
A (MSH2)	X (ERCC1)	S179 F722 M723 M726 A730 L733 K734 T751 S752 Y754 D755 F757 G758 L759 W761 A762 I763 E765 H766 Q769 V770 K771 E783 A786 Q789 A790 N791 S792 E793 V794 L849 E852 K853 E856 L857 D859 F860 A889 E890 H893 L896 K897
X (ERCC1)	A (MSH2)	N124 N143 V144 K145 K190 L191 D199 E201 D202 T203 V204 L207 L208 T211 K212 L215 L216 D218 C219 T220 L221 L222 C223 W225 E229 R232 Y233 T236 I237 V239 Y240 E241 N242 P244 A245 L247 I248 Q251
**Chain**	**Interacting chain**	**Residues**
A (MSH2)	X (MLH1)	E216 G666 P667 N668 C714 Q715 L716 R717 R749 G750 T751 S752 T753 Y754 H780 H782 E783 R815 S831 F832 G833 I834 V836 A837 F839 A840 N841 F842 P843 S845 V846 A848 L849 A850 E852 K853 A854 E856 L857 F860
X (MLH1)	A (MSH2)	L32 N38 A41 V45 D62 A63 D64 H87 R90 E92 D93 R100 H101 S104 K105 L106 K108 F109 E110 D111 L112 F113 S114 L115 S116 M118 G119 F120 E123 S127 Y130 H326 V327 I329 N330 P333 T334 K335 K336 V338 S339 Q343
**Chain**	**Interacting chain**	**Residues**
A (MSH2)	X (MLH3)	Y754 D755 F757 G758 W761 A762 E765 Q769 E783 A786 Q789 A790 N791 S792 E793 N797 K853 A855 E856 D859 F860 S861 P882 D883 V885 S886 A889 E890 A892 H893 L896 K897 A900 W932 F936
X (MLH3)	A (MSH2)	L7 P8 V11 R12 M15 R16 G18 I19 F22 D23 R26 G48 V49 S51 C52 S53 R74 R96 K130 V158 R159 R160 K161 Y162 M163 Q164 S165 S166 P167 K168 K169 E172 R180
**Chain**	**Interacting chain**	**Residues**
A (MSH2)	X (PCNA1)	R717 S752 T753 Y754 D755 E856 L857 D859 F860 S861 V885 S886 A889 A892 H893 F895 L896 K897 F899 A900 L904 D905 K920 L923 W932 L933 F936 L937
X (PCNA1)	A (MSH2)	R138 V159 K164 E165 K168 S170 A172 D174 I175 G176 T177 N179 V181 R183 Q184 N185 T186 T187 V188 D189 P191 A194 V196 E198 E243
**Chain**	**Interacting chain**	**Residues**
A (MSH2)	X (PCNA2)	D34 T35 R36 E52 S179 R180 S205 N206 K209 S210 D213 R217 L716 R717 G718 V719 S720 F722 M723 Q724 M726 L727 A730 K734 T751 S752 D755 L759
X (PCNA2)	A (MSH2)	T32 M40 S43 H44 V45 L47 D63 R64 N65 E124 L126 G127 I128 P129 D130 A131 E132 H134 R138 N200 E201 L205 S206

		S231 E232 L233 P234 R248 Y250 A252 P253
**Chain**	**Interacting chain**	**Residues**
A (MSH2)	X (PMS1)	Y754 D755 F757 G758 W761 E765 H766 Q769 V770 A790 E856 L857 E858 D859 S861 V885 S886 A889 E890 A892 H893 F895 L896 K897 F899 A900 L904 D905 V916 K920 W932 L933 F936 L937
X (PMS1)	A (MSH2)	I20 N21 R22 N23 V24 I25 C29 K88 H89 H90 T91 S92 L94 E95 L101 T104 E111 H141 K163 S166 N167 P169 V170 K173 R177 P323
**Chain**	**Interacting chain**	**Residues**
A (MSH2)	X (RECQSIM)	F151 H152 D153 G154 E174 S205 N206 K209 D213 E216 K231 R233 G256 D258 D713 C714 Q715 L716 V719 S720 M723 Q724 M726 L727 L733 K734 T751 Y754 D755 F757 G758 L759 W761 A762 I763 E765 H766 E783 A786 E858 D859 F860 S861 P882 D883 V885 S886
X (RECQSIM)	A (MSH2)	D602 L603 P607 L610 R614 F630 M634 T636 H697 Y700 K709 Q710 K712 L713 S714 H715 K716 P717 N718 L719 L720 F721 F722 S724 K742 D758 N759 D761 R762 R763 V764 E765 I766 K767
**Chain**	**Interacting chain**	**Residues**
A (MSH2)	X (TIL1)	T311 D312 N668 M669 S673 A711 G712 C714 Q715 L716 R717 G718 V719 T721 F722 D745 E746 R749 G750 T751 S752 T753 Y754 D755 H780 G833 I834 V836 A837 A840 F842 E844 S845 V846 L849 A850 K853 A854 E856 L857 F860 K920 E924 L937
X (TIL1)	A (MSH2)	M98 E99 E101 A104 Y105 R108 R109 H158 E161 R162 Q164 A165 K166 F167 D168 A169 L170 E171 A172 Y173 E174 S175 I176 L177 A178 G179 K180 D187 C188 L189 K659 E663 M674 S675 S676 K684 Q687
**Chain**	**Interacting chain**	**Residues**
A (MSH2)	X (UVH1)	S752 T753 Y754 D755 F757 E783 A786 E856 L857 D859 F860 S861 S886 A889 E890 A892 H893 F895 L896 K897 F899 A900 I902 P903 L904 D905 L909 S912 L913 V916 R917 K920 L923 W932 L933 F936 L937
X (UVH1)	A (MSH2)	F733 M734 S735 S736 N739 H742 Q743 K744 P750 F803 S804 D806 S808 F809 Y824 I827 L830 S831 V834 L835 H836 L842 L843 W844 S845 R846 S847 L848 H849 A850 E853 T856 T857
**Chain**	**Interacting chain**	**Residues**
A (MSH3)	X (At1G18090)	L50 R130 R132 E139 A142 R143 V144 G146 I147 Y148 A149 H150 M151 D152 M156 P161 F163 R164 N166 F167 H168 R170 R171 C224 E227 Q668 V669 S670 I672 V689 K690 V691 N692 S693 T694
X (At1G18090)	A (MSH3)	R8 P12 I14 L15 P16 E165 L166 N206 F208 V211 Q213 S216 F217 Q218 F220 D221 Q222 E223 L224 Q256 S257 V258 E259 L260 K266 V285 Q289 L304 S308 H309 L311 L312 N313
**Chain**	**Interacting chain**	**Residues**
A (MSH3)	X (At1G29630)	E93 Y94 V95 P96 R102 K103 T105 P106 L107 E108 V127 G128 Y129 R130 R132 H150 M151 D152 H153 N154 P161 F163 A188 A189 K191 S192 R197 G199 P200 F201 R203 F540 V669 S670 G671 I672 W688 P702 V706 L709 D710 A713 E717
X (At1G29630)	A (MSH3)	I3 L6 L7 L9 L10 K11 S12 I13 M14 V15 P16 I17 H18 K20 E21 I26 Y58 H61 L65 L82 P83 V145 P148 Y149 A153 Q154 F157 I160 T161 K162 Q163 D165 I175 P176 F177 G178 C179 L180 R181
**Chain**	**Interacting chain**	**Residues**
A (MSH3)	X (At2G02550)	L50 R130 R132 E139 I140 A142 R143 V144 L145 G146 I147 Y148 A149 H150 M151 N154 M156 R164 F167 R171 A175 Y177
X (At2G02550)	A (MSH3)	F48 L49 E50 P52 N56 S57 R85 P94 I95 Y100 K102 D103 L104 A106 S107 A108 L110 M111 D114 R118 H132 H134 T136 P137 Y138 V139 V142

Chain	Interacting chain	Residues
A (MSH3)	X (ERCC1)	Y129 R130 P161 T162 F163 R164 N166 F167 G223 C224 G226 E227 V511 R542 T543 A544 T547 I550 A551 E554 L558 K561 R565 V616 D619 L620 L621 V643 K647 E665 L667 Q668 V669 S670 I672 T673 L712 A713 T716
X (ERCC1)	A (MSH3)	R142 N160 H171 P175 D176 Y179 R183 K190 L191 V198 D199 E201 D202 T203 V204 K205 L207 L208 E209 K212 T213 A214 L215 L216 D218 C219 T220 L221 L222 C223 W225 S226 T228 E229 R232 Y233 T236 V239 Y240 E241 N242 K243 L247
**Chain**	**Interacting chain**	**Residues**
A (MSH3)	X (MLH1)	F163 R164 T543 T547 V616 R617 D619 L620 L621 L624 S627 E636 A637 A640 V641 V643 I644 K647 L648 S651 R662 N663 E665 F666 L667 Q668 V669 S670 I672 T673 H674 E677 S693 T694 K695 K696 L712 L719
X (MLH1)	A (MSH3)	E44 Q47 L106 E110 D111 F113 S114 L115 S117 M118 F120 D258 S260 G261 C293 S294 A295 R298 L322 R324 H326 I329 N330 P333 T334 K335 E337 V338 S339 L340 L341 N342 Q343 E344 I345 I347 E348 E353
**Chain**	**Interacting chain**	**Residues**
A (MSH3)	X (MLH3)	E248 T249 L250 C252 G253 I254 E255 F258 R261 E277 E278 N280 N282 L355 E356 K359 K362 L363 A365 A366 G369 M370 S371 M379 N380 M381 R412 S413 S415 S416 N417 T418 E419 T421 Y456 L460 H463 W464 H467 D471 R472 N473 L474 A477 R478 R583 S584 L586 L590 K742 Q746 A750 D774 D854 R885 T886 S888 R890
X (MLH3)	A (MSH3)	L7 P8 V11 R12 M15 R16 G18 I19 F22 D23 R26 E29 F33 R65 D66 D67 V69 L70 E73 Y75 A76 T77 S78 F80 H81 D82 F83 T84 N85 V86 E87 T88 A89 S90 F93 F95 R96 E98 Q156 P157 V158 R159 Y162 K169 S173 K176 R180 L183 V184
**Chain**	**Interacting chain**	**Residues**
A (MSH3)	X (PCNA1)	Y129 R130 R132 L145 G146 I147 Y148 H150 S159 P161 F163 R164 N166 F167 R171 S221 G223 C224 E227 E507 I510 V511 T543 A544 T547 D619 L620 L621 K647 Q668 V669 S670 T673 V691 N692 S693 T694
X (PCNA1)	A (MSH3)	E17 K20 D21 N24 M40 S42 S43 H44 V45 L47 K77 K80 D122 E124 H125 L126 G127 I128 P129 D130 A131 E132 H134 D156 V159 K164 K168 S170 A172 G173 D174 T177 N179 K200 E201 S204 L205 S206 R210 Y211 S214 E232 L233 P234 A252 P253
**Chain**	**Interacting chain**	**Residues**
A (MSH3)	X (PCNA2)	R130 R132 L145 G146 I147 Y148 A149 H150 M151 S159 P161 F163 R164 F167 H168 R171 T543 D619 L620 L621 L667 Q668 V669 S670 V691 S693 T694
X (PCNA2)	A (MSH3)	Q8 D29 S31 T32 T33 Q38 L47 S54 E55 G56 F57 E58 Y60 R61 D63 R64 N65 D94 D122 E124 H125 L126 G127 I128 P129 D130 A131 Y133 H134 E232 L233 P234 Y250
**Chain**	**Interacting chain**	**Residues**
A (MSH3)	X (PMS1)	K183 Q184 T185 E186 T187 A189 I190 H193 G194 A195 F202 G204 L205 S345 L346 K397 R403 Q533 G535 I536 T537 R538 I539 F540 Q630 F631 P632 E633 A635 R638 Q639 L642 R645 D649 D710 E711 L714 E717 H718 I721 V722 A725
X (PMS1)	A (MSH3)	I20 N21 R22 N23 V24 I25 R27 I28 Q32 L35 D36 K88 H89 H90 T91 E95 L100 H141 T146 K163 S166 N167 L168 P169 V170 R171 K173 E174 R177 T322 P323 K325
**Chain**	**Interacting chain**	**Residues**
A (MSH3)	X (RecQSIM)	E93 Y94 V95 P96 T98 R102 K103 T105 P106 L107 M151 H153 E186 A188 A189 I190 K191 S192 H193 G194 A195 N196 R197 G199 P200 F202 Q533 E633 L634 A635 R638 Q639 L642 R645 D649 V691 N692 P703 A707 D710 E711 L714 H718
X (RecQSIM)	A (MSH3)	V369 K370 H371 R373 K375 K508 A601 D602 L603 A606 P607 L610 P611 R613 R614 E620 Y623 K624 S627 F630 R631 G633 M634 T636 R640 E665 N707 Q710 E735 T736 D737 C738 L739 K760 E765 V789 P791 E792 A793 D794 M795 L796 L797

**Chain**	**Interacting chain**	**Residues**
A (MSH4)	X (MLH1)	R83 G553 P554 N555 N601 L602 E603 S604 N605 R636 A637 T638 S639 S640 S641 H666 M667 D668 S669 H709 G711 L712 L714 A715 A718 G719 L720 P721 S722 T723 V724 D726 T727 A728 I730 I731 T732 K733 R734 I735 D737 K738 Y757
X (MLH1)	A (MSH4)	L32 N38 A41 E44 V45 D62 A63 D64 H87 G88 R90 D93 R100 H101 S104 K105 L106 K108 F109 E110 D111 L112 F113 S114 L115 S116 S117 M118 G119 F120 E123 S260 E325 H326 V327 I329 N330 P333 T334 K335 E337 V338 S339 N342 Q343
Chain	Interacting chain	Residues
A (MSH4)	X (MLH3)	Q621 S640 S641 D642 L644 A645 M646 W648 S649 E652 S656 S669 E672 T675 I676 Y677 R734 I735 D737 E739 I743 E744 N746 C747 H750 H751 I753 H754 R755 Y757 R758 Q761
X (MLH3)	A (MSH4)	R12 M15 R16 G18 I19 F22 D23 R26 E29 D66 V69 L70 E73 R74 Y75 A76 T77 S78 F80 H81 F83 V86 E87 R96 Q156 V158 R159 Y162 K169 R180
**Chain**	**Interacting chain**	**Residues**
A (MSH4)	X (MUS81)	H182 N183 T201 T202 K203 T204 A205 Q247 L249 R250 K251 P253 E255 T256 R258 K269 I287 S288 L292 T295 A296 D298 A299 V343 L344 H345 A346 R347 V348 P349 F350 A352 R353 T354 Q355 Q356 C357 F358 A359 N397 L398 K399 P401 F402 N403 R411 P413 T460 E461 L464
X (MUS81)	A (MSH4)	D408 R410 E411 K412 F413 A414 T415 R439 P441 D444 H451 K452 Y453 L454 E465 R473 S474 I476 R477 D478 N479 R480 R482 D483 Q484 L486 R487 R490 H506 S507 D508 A509 A510 S512 I513 T515 A516 T519 R577 C578 Q579 D580 L581 D582 M584 I592 T601 E602
**Chain**	**Interacting chain**	**Residues**
A (MSH4)	X (PMS1)	S641 D642 A645 W648 S649 E652 E672 T675 I676 R734 I735 D737 K738 I743 N746 C747 G748 H750 H751 I753 H754 R755 Y757 R758 A760 Q761 R762 I764 C765 K767 R770 S786 E789 E790
X (PMS1)	A (MSH4)	I20 N21 R22 V24 I25 I28 C29 Q32 E43 E46 N47 D50 A51 C75 G76 I77 S78 N81 L85 H89 T91 S92 L100 L101 T105 R109 G110 E111 L113 R127 T128 N130 I155 N278 R280 G314 G315 C317 L319 N320 V321 T322 P323 R326
**Chain**	**Interacting chain**	**Residues**
A (MSH4)	X (RAD51)	A71 P554 N555 S604 N605 S606 F609 M610 M613 A617 M620 Q621 R636 A637 T638 S639 S640 S641 D642 L644 A645 M646 W648 S649 E652 Y653 S656 H666 E672 T675 I676 H709 G711 L712 L714 T727 A728 I731 T732 I735 K738 Y757
X (RAD51)	A (MSH4)	F132 R133 E166 G167 T168 R170 R196 Y198 N199 A229 R232 T233 D234 F235 S236 G237 R238 E240 Q271 V272 V273 V276 G278 S279 A280 L281 F282 A283 G284 Q286 F287 P289 K307 E327
**Chain**	**Interacting chain**	**Residues**
A (MSH4)	X (SPO11-1)	K733 R734 E752 I753 I756 Y757 V759 A760 Q761 R762 L763 I764 C765 L766 K767 Y768 S769 I776 A779 L780 N782 L783 N784 E785 S786 F787 T788 E789
X (SPO11-1)	A (MSH4)	Q72 Y74 R81 V82 L83 L84 I85 V86 Q87 Q88 L89 K98 I101 Y102 M104 H105 S107 A108 A111 Q112 I114 V115 D116 R117 A118 I119 L125 F126
**Chain**	**Interacting chain**	**Residues**
A (MSH5)	X (MLH1)	G569 P570 N571 Y572 K615 M617 T618 A619 E620 Q621 K651 G652 T653 L654 T655 E656 H683 T685 E686 S733 G735 L736 C738 A739 A742 G743 V744 P745 E747 V748 R751 A752 I754 V755 L756 A758 F759 S761 N762 K781
X (MLH1)	A (MSH5)	E34 I40 A41 E44 V45 D62 A63 D64 H87 R90 R100 S104 L106 F109 E110 D111 L112 F113 S114 L115 S116 M118 F120 S260 R324 E325 H326 V327 I329 N330 P333 T334 K335 K336 V338 S339 N342 Q343

Chain	Interacting chain	Residues
A (MSH5)	X (MLH3)	R22 R23 E40 F41 W42 E43 E44 C46 L51 E72 F74 M132 D133 S136 M175 E176 P178 N180 K181 K184 L241 D242 L243 V530 E531 S532 D560 N561 L604 D606 T639 S640 R641 T671 C672 E674 P675 Q12
X (MLH3)	A (MSH5)	L7 E9 R12 M15 R16 G18 I19 F22 D23 R26 R74 Y75 F80 F83 V86 E87 F95 R96 F153 Q156 P157 V158 R159 K161 Y162 M163 Q164 K168 K169 E172 S173 K176 R180
**Chain**	**Interacting chain**	**Residues**
A (MSH5)	X (PCNA1)	V112 T113 D116 L119 N120 I121 S166 I169 A170 I171 N284 E333 T341 E342 Y345 E348 L349 G371 F372 C373 A374 E375 D377 I483 D486 S489 H490 L492 L493 F494 S495 A496 H497 L499
X (PCNA1)	A (MSH5)	R138 E143 R146 K149 D150 S153 I154 T163 K164 E165 K168 I175 A178 N179 I180 V181 L182 R183 Q184 N185 T186 T187 V188 D189 P191 A194 I195 V196
**Chain**	**Interacting chain**	**Residues**
A (MSH5)	X (PCNA2)	I207 G208 R209 T291 N293 R358 K360 E361 R362 G363 Y364 Q365 K408 P410 P411 V414 Y415 I416 Q417 Q418 C424 F426 G427 L430 D431 Q453 R454 Y473 R481
X (PCNA2)	A (MSH5)	D29 S31 T32 T33 H44 S54 E55 F57 E58 H59 Y60 R61 C62 D63 N65 T107 Q108 D109 D122 S123 E124 H125 L126 G127 P129 D130 A131 E232 L233
**Chain**	**Interacting chain**	**Residues**
A (MSH5)	X (PMS1)	N571 Y572 K651 L654 T655 E656 I659 H683 E686 S733 G735 L736 C738 A739 A742 G743 V744 P745 E747 V748 K750 R751 A752 I754 V755 L756 A758 F759 S761 N762 D785
X (PMS1)	A (MSH5)	N21 R22 N23 I25 H26 C29 E46 N47 D50 K88 H89 H90 T91 S92 K93 L94 E95 D96 T98 L100 L101 T104 T105 F108 R109 G110 E111 L144 V170 N320 P323
**Chain**	**Interacting chain**	**Residues**
A (MSH5)	X (RECQ4A)	R358 E361 R362 Y364 E388 Q391 E392 A395 M396 E397 L398 H400 H403 L404 H405 K406 E407 K408 L409 P410 P411 C412 V414 Y415 I416 Q417 Q418 Y421 C424 I425 F426 G427 E428 K429 L430 D431 E432 T433 A434 L435 N436 R437 L438 E440 E442 F443 Q453 R454
X (RECQ4A)	A (MSH5)	S431 R433 K446 N451 H452 S453 F454 R455 P456 N457 R459 E460 G478 G479 Q641 S642 N644 P646 W649 E669 N670 G768 Q769 R770 D847 S848 T849 N850 C851 K852 K853 T854 C858 C859 Q862 S863 L864 D866 S987 K988 Q989 G990 A991 T992 P1014 P1015 D1017 V1018 S1052 Q1056 R1059 R1060 R1063 T1064 K1065 E1066 E1067 E1070 N1072 Y1097
**Chain**	**Interacting chain**	**Residues**
A (MSH5)	X (RECQ4B)	V112 T113 M115 D116 G118 L119 N120 I121 S166 I169 A170 I171 S173 N284 T341 D344 Y345 E348 L349 G352 F372 C373 I483 D486 S489 H490 L492 L493 F494 S495 A496 H497 L499
X (RECQ4B)	A (MSH5)	V887 R890 Q891 A894 L895 K897 L898 R902 F903 H907 S919 K921 R922 N923 R924 Q925 D926 T927 L928 H929 A933 K960 R976 A980 L983 S984
**Chain**	**Interacting chain**	**Residues**
A (MSH5)	X (RECQI1)	T113 D116 L119 N120 K122 R321 R325 L329 I331 I332 E333 L337 C338 T341 E342 D344 Y345 E348 L349 I351 G352 G371 F372 C373 A374 D486 S489 H490 L493 F494 A496 H497
X (RECQI1)	A (MSH5)	F207 R208 Q211 H212 R216 M219 E220 G233 S235 L236 Q239 L240 T243 L244 Q262 V264 N267 L268 K269 F270 G271 I272 T275 F276 S284 Q285 A288 D427 Q428 Q475 A495 D496 R498 D522 L524
**Chain**	**Interacting chain**	**Residues**
A (MSH5)	X (RECQI3)	Y100 W104 H105 V112 T113 M115 D116 D117 L119 N120 K122 R124 I125 C126 Y127 S129 E137 S164 S166 I169 A170 N284 E333 E342 D344 Y345 E348 L349 D486 S489 H490 L492 L493 F494 S495 H497

X (RECQI3)	A (MSH5)	L24 V25 K26 R29 W30 G33 H34 A35 D36 F37 R38 K40 L42 E43 Q46 A47 V49 S50 R52 L219 K222 N226 P228 R326 D350 Q351 L352 P353 Y523 R525 N526 V529 K530
**Chain**	**Interacting chain**	**Residues**
A (MSH5)	X (RECQSIM)	E101 Q102 W104 H105 R106 V108 Y109 I121 K122 E123 R124 I125 C126 S129 S130 M132 E153 I155 E163 S164 G165 S166 I169 A170 F283 N284 S285 P286 S288 L289 C290 L488 S489 L492 L493
X (RECQSIM)	A (MSH5)	V691 D692 N693 S695 E696 H697 P699 Y700 Y703 K709 Q710 N711 K712 L713 S714 H715 P717 N718 L719 L720 F721 F722 S724 R727 C738 K742 N759 D761 R762 V764 I766
**Chain**	**Interacting chain**	**Residues**
A (MSH6)	X (MLH1)	R713 N714 R1060 N1085 M1086 I1132 M1133 A1134 G1135 R1166 G1167 T1168 A1169 T1170 Q1174 H1197 Y1198 H1199 R1200 I1224 G1225 G1226 V1227 G1246 V1249 A1250 L1252 A1253 G1254 L1255 P1256 Y1258 V1259 R1262 A1263 K1266
X (MLH1)	A (MSH6)	E34 V37 N38 A41 V45 R100 H101 L106 F109 E110 L112 F113 S114 L115 M118 F120 E123 G261 C262 R324 E325 H326 I329 N330 P333 T334 K335 K336 E337 V338 N342 Q343 E344 I345
**Chain**	**Interacting chain**	**Residues**
A (MSH6)	X (MLH3)	L770 R773 F1018 Y1023 E1024 G1025 V1026 T1046 G1047 P1051 L1053 R1054 L1058 G1059 R1060 G1061 S1062 F1063 V1064 P1065 N1067 E1112 T1113 E1115 C1218 Q1219 I1224 E1228 E1229 V1230 F1232 L1233 R1235 T1237
X (MLH3)	A (MSH6)	L7 P8 M15 R16 G18 I19 F22 R26 E29 R74 Y75 T77 F80 D82 F83 V86 E87 T88 S90 F95 R96 E98 Q156 P157 V158 R159 K161 Y162 S173 K176 R180
**Chain**	**Interacting chain**	**Residues**
A (MSH6)	X (PCNA1)	R345 V349 R351 R358 P359 T360 Y364 P366 M394 D395 K418 K449 G450 Y451 R452 P520 A568 Y573 A574 E576 R577 V580 R581 R584 N585 P586 L587 V588 N590 H1131 Q1136 S1137 T1138 F1139 L1140 T1141 G1167 T1168
X (PCNA1)	A (MSH6)	M1 E3 R5 N27 D29 S31 S32 T33 S36 Q38 M40 S42 S43 H44 L51 S54 E55 E58 H59 Y60 R61 C62 D63 R64 N65 L66 S67 D93 D94 G95 Q108 D120 I121 D122 S123 E124 H125 L126 G127 P129 D130 A131
**Chain**	**Interacting chain**	**Residues**
A (MSH6)	X (PCNA2)	V349 R351 R358 P359 T360 Y364 P366 M394 D395 K418 Y451 R452 P520 K566 A568 Y573 E576 R577 V580 R581 R584 N585 P586 L587 V588 N590 Q1136 S1137 T1138 F1139 L1140 T1141 T1168
X (PCNA2)	A (MSH6)	M1 E3 R5 N27 D29 S31 T32 T33 S36 Q38 M40 H44 S54 E55 E58 H59 Y60 R61 C62 D63 R64 N65 L66 S67 D93 D120 I121 D122 S123 E124 H125 L126 G127 P129 A131
**Chain**	**Interacting chain**	**Residues**
A (MSH6)	X (PMS1)	Q727 A759 R762 G763 E764 N765 L766 P767 L770 R773 K774 F1018 D1021 S1022 Y1023 E1024 G1025 V1026 R1027 C1028 R1029 G1047 L1053 R1054 G1055 L1058 G1059 G1061 V1064 P1065 E1112 L1233 R1235
X (PMS1)	A (MSH6)	I20 R22 I25 R27 I28 C29 G31 Q32 V33 L35 D36 E43 N47 D50 K88 H89 H90 T91 S92 L94 E95 L100 T104 F108 R109 G110 E111 L168 V170 R171 N320 V321 T322 P323
**Chain**	**Interacting chain**	**Residues**
A (MSH6)	X (RECQSIM)	E707 S712 R713 N714 G715 Y717 G719 H1050 V1052 G1055 D1056 S1057 R1060 S1062 F1063 N1066 M1086 G1088 S1090 T1091 R1094 L1098 D1130 I1132 M1133 A1134 G1135 R1166 E1222 G1223 I1224 G1225 G1226 V1227 E1228 F1232 L1233 Y1234 N1248 V1249 A1250 R1251 L1252 A1253 G1254 L1255 P1256 D1257 Y1258 Q1261 R1262
X (RECQSIM)	A (MSH6)	R163 I218 S219 H222 D223 L226 S229 R230 K232 V233 S234 A235 C236 F237 G239 S240 G241 Q242 L243 D244 N245 C246 I247 E248 E249 M252 Q253 G254 M255 Y256 Q257 V266 V267 R268 K271 K275 L276 K278 T279 E497 R537

		H540 Q541 H544 D545 N546 K563 K564 N565
**Chain**	**Interacting chain**	**Residues**
A (MSH6)	X (TIL1)	T518 N519 P520 S572 Y573 E576 R577 V580 T1082 G1083 P1084 N1085 I1132 M1133 G1135 S1137 R1166 G1167 T1168 A1169 T1170 S1171 H1197 H1199 S1202 A1240 C1241 K1243 S1244 Y1245 G1246 V1247 V1249 A1250 A1253 L1255 P1256 Y1258 V1259 A1263 K1266 S1267 F1270
X (TIL1)	A (MSH6)	M98 E99 E101 R162 A165 K166 F167 D168 A169 L170 E171 A172 Y173 E174 S175 I176 L177 A178 R184 P185 Q186 D187 L189 H657 K660 Q661 S664 E665 F666 V667 D668 S676 K677 F679 D681
**Chain**	**Interacting chain**	**Residues**
A (MSH6)	X (TIL2)	T518 N519 P520 S572 Y573 E576 R577 V580 T1082 G1083 P1084 N1085 I1132 M1133 G1135 S1137 R1166 G1167 T1168 A1169 T1170 S1171 H1197 H1199 S1202 A1240 C1241 K1243 S1244 Y1245 G1246 V1247 V1249 A1250 A1253 L1255 P1256 Y1258 V1259 A1263 K1266 S1267 F1270
X (TIL2)	A (MSH6)	M98 E99 E101 R162 A165 K166 F167 D168 A169 L170 E171 A172 Y173 E174 S175 I176 L177 A178 R184 P185 Q186 D187 L189 H657 K660 Q661 S664 E665 F666 V667 D668 S676 K677 F679 D681
**Chain**	**Interacting chain**	**Residues**
A (MSH7)	X (MLH1)	L465 P854 N855 D900 R901 I902 M903 T904 E906 R936 T938 S939 T940 F941 H967 P970 G1021 L1022 V1024 A1025 A1028 G1029 I1030 P1031 Q1033 V1034 E1036 T1037 A1038 G1040 A1041 A1044 M1045 R1047 S1048 E1051
X (MLH1)	A (MSH7)	L32 N38 A41 E44 V45 D62 A63 D64 H87 R90 R100 H101 S104 L106 K108 F109 L112 F113 S114 L115 S117 M118 G119 F120 E123 A124 S127 G261 T263 H326 V327 I329 N330 P333 T334 K335 V338 S339 L340 L341 N342 Q343
**Chain**	**Interacting chain**	**Residues**
A (MSH7)	X (MLH3)	R418 Y497 F498 K499 G500 S501 S504 N506 C507 A508 D510 I543 P545 Y546 Q547 R550 C552 L593 L602 D604 E639 M695 C703 K704 L705 I707 L708 V709 G710 S712 G713 E715 L716 F717 Q720 E750 L751 E754 R755 T757 Q758 S760 E761 H764 C768
X (MLH3)	A (MSH7)	L7 E9 R12 M15 R16 G18 I19 I20 R26 F33 D66 V69 L70 E73 R74 Y75 A76 T77 S78 F80 H81 D82 F83 T84 N85 V86 E87 T88 S90 E91 F93 F95 R96 E98 L132 H133 L134 I136 R180 V184
**Chain**	**Interacting chain**	**Residues**
A (MSH7)	X (PCNA1)	E232 W233 R238 R240 N243 R244 R245 R246 D249 P250 L251 D253 H258 P260 P261 K265 K450 L473 A474 P475 V476 P477 M480 D482 T483 A486 R531
X (PCNA1)	A (MSH7)	M1 D29 S31 S32 T33 S36 Q38 S49 L51 R53 S54 E55 H59 Y60 R61 C62 D63 N65 E124 H125 L126 G127 I128 P129 D130 A131 E132 Y246 R248 Y250
**Chain**	**Interacting chain**	**Residues**
A (MSH7)	X (PCNA2)	D823 G824 Q825 L826 V828 P829 P854 N855 M856 G857 L1005 V1006 F1007 L1008 Y1009 R1010 Q1023 V1024 M1027 A1028 G1029 I1030
X (PCNA2)	A (MSH7)	S31 T33 S36 Q38 S49 L51 R53 S54 E55 L126 G127 I128 P129 D130 A131 E132 Y133 I136 R138 T224 S228 E232 L233 P234 V236 K240 E243 Y246 R248 Y250 P253
**Chain**	**Interacting chain**	**Residues**
A (MSH7)	X (PMS1)	R454 I902 T904 G905 F909 L910 C913 T914 T938 S939 T940 F941 D942 Y944 A945 I946 Y948 S949 P970 E974 A1041 A1044 M1045 R1047 S1048 I1049 E1051 N1052 F1053
X (PMS1)	A (MSH7)	I20 N21 R22 N23 V24 I25 H26 R27 I28 C29 Q32 K88 H89 H90 T91 S92 K93 E95 D99 L100 T104 E111 L168 V170 R171 E174 N320 V321
**Chain**	**Interacting chain**	**Residues**
A (MSH7)	X (RECQSIM)	R418 W420 D482 A485 Y497 F498 S504 W505 N506 C507 A508 D510 G511 L512 N513 E514 G541 I543 P545 Y546 Q547 V548 R550 G551 C552 L593 N596 D604 E606 S607 R611 K646 M695 L698 C703 I707 L708 V709 E754 A756

		T757 Q758 S760 E761 H764 C768 K803
X (RECQSIM)	A (MSH7)	K154 R155 V156 N157 R174 S178 T179 V181 A182 H183 K184 T207 G208 K209 Q274 K278 H280 V309 E312 N313 C315 S317 N318 L319 E320 F321 L322 E323 Y324 K351 E352 K354 I355 V356 L357 T358 S359 F360 F361 R362 E651
**Chain**	**Interacting chain**	**Residues**
A (MSH7)	X (TIL1)	A387 R418 W420 F498 K499 N506 C507 A508 D510 E514 I543 P545 Y546 Q547 V548 R550 N596 L699 C703 K704 I707 L708 V709 G710 S712 G713 L716 F717 T757 Q758 S760 E761 H764 T765
X (TIL1)	A (MSH7)	M98 E99 L100 E101 A104 Y105 R108 K166 F167 A169 L170 E171 A172 Y173 E174 S175 I176 L177 A178 K180 R184 P185 Q186 D187 C188 L189 E621
**Chain**	**Interacting chain**	**Residues**
A (MSH7)	X (TIL2)	A416 R418 F498 S501 S502 N506 C507 A508 D510 I543 P545 Y546 Q547 R550 L593 E606 K610 E639 K646 S697 L698 C703 P706 I707 L708 V709 G710 S712 R755 T757 Q758 S760 E761 I763 H764
X (TIL2)	A (MSH7)	D88 N89 M90 E91 E93 K158 F159 N160 A161 L162 E163 A164 Y165 E166 S167 I168 L169 Q175 P177 Q178 D179 C180 L181 D182 K651 E655
